# Reward circuitry dysfunction in psychiatric and neurodevelopmental disorders and genetic syndromes: animal models and clinical findings

**DOI:** 10.1186/1866-1955-4-19

**Published:** 2012-07-06

**Authors:** Gabriel S Dichter, Cara A Damiano, John A Allen

**Affiliations:** 1 Carolina Institute for Developmental Disabilities, University of North Carolina at Chapel Hill School of Medicine, Chapel Hill, NC, USA; 2 Department of Psychiatry, University of North Carolina at Chapel Hill School of Medicine, Chapel Hill, NC, USA; 3 Department of Psychology, University of North Carolina at Chapel Hill, Chapel Hill, NC, USA; 4 Neuroscience Research Unit Pfizer Global Research and Development, Groton, CT 06340, USA; 5 Department of Psychiatry, University of North Carolina School of Medicine, CB# 7255, 101 Manning Drive, Chapel Hill, NC, 275997255, USA

**Keywords:** Reward, Mesolimbic, Dopamine, Nucleus Accumbens, Striatum, Neuroimaging

## Abstract

This review summarizes evidence of dysregulated reward circuitry function in a range of neurodevelopmental and psychiatric disorders and genetic syndromes. First, the contribution of identifying a core mechanistic process across disparate disorders to disease classification is discussed, followed by a review of the neurobiology of reward circuitry. We next consider preclinical animal models and clinical evidence of reward-pathway dysfunction in a range of disorders, including psychiatric disorders (i.e., substance-use disorders, affective disorders, eating disorders, and obsessive compulsive disorders), neurodevelopmental disorders (i.e., schizophrenia, attention-deficit/hyperactivity disorder, autism spectrum disorders, Tourette’s syndrome, conduct disorder/oppositional defiant disorder), and genetic syndromes (i.e., Fragile X syndrome, Prader–Willi syndrome, Williams syndrome, Angelman syndrome, and Rett syndrome). We also provide brief overviews of effective psychopharmacologic agents that have an effect on the dopamine system in these disorders. This review concludes with methodological considerations for future research designed to more clearly probe reward-circuitry dysfunction, with the ultimate goal of improved intervention strategies.

## Review

### Introduction

Despite the categorical nosology of the *Diagnostic and Statistical Manual of Mental Disorders* (DSM) [1], different neurodevelopmental and psychiatric disorders share phenotypic features, etiologies, and aberrant neurobiological processes. Indeed, there are multiple examples of distinct disorders that are characterized by common pathophysiological mechanisms. For example, anxiety disorders and mood disorders share hyperactive amygdala responses to negatively valenced stimuli [2,3] and schizophrenia and post-traumatic stress disorder are both characterized by prefrontal dysfunction during tasks that require sustained attention [4,5]. Such overlap suggests the utility of examining common patterns of dysregulated brain function and associated phenotypes with the ultimate goal of more accurately linking pathophysiological processes to rationally derived and targeted interventions.

The identification of common neurobiological deficits across disparate neurodevelopmental and psychiatric disorders has helped to motivate goal 1.4 of the NIMH Strategic Plan [6], the Research Domain Criteria project (RDoC; http://www.nimh.nih.gov/research-funding/rdoc.shtml), which aims to foster research that uses neuroscience tools to investigate constructs that cut across traditional nosological classification boundaries [7,8]. Although optimal methodological approaches to address these questions are still emerging, the ultimate goal of this framework is to refine classification and develop empirically derived approaches to treatment [9-11]. At the heart of this approach is the search for dysfunctional mechanistic processes shared by disorders with seemingly disparate phenotypic profiles, a strategy that represents a particular instantiation of the endophenotypic approach to identifying pathophysiological disease mechanisms [12-14].

The functioning of reward-processing systems through development has recently garnered increased research attention in both nonclinical [15,16] and clinical [17-19] contexts, and the functioning of so-called ‘positive valence systems’ has been proposed as one of the five domains relevant to the NIMH RDoC project [6]. Given the focus of this thematic issue on reward processing in autism specifically, the purpose of this review is to place dysfunctional reward processing in autism within the larger context of emerging evidence that reward-circuitry dysfunction may be present in multiple distinct disorders, and may thus represent a common target for treatments of these disorders.

In this review, we summarize preclinical models and clinical research addressing reward-circuitry dysfunction in a range of neurodevelopmental and psychiatric disorders and genetic syndromes. Specifically, we focus on the functional output of ascending mesolimbic dopamine (DA) projections systems, referred to broadly in this review as ‘reward-processing’ systems. In its fundamental unit, the mesolimbic DA pathway consists of a population of DA-containing neurons in the ventral tegmental area (VTA) that project to neurons in the nucleus accumbens (NAc); however, these VTA neurons also extend projections into the amygdala, the bed nucleus of the stria terminalis, the lateral septal area, and the lateral hypothalamus (collectively, these connections comprise the entire mesolimbic DA system). The processes subserved by these systems have been referred to by multiple names in the research literature, including ‘motivation’ [20], ‘goal-directed behaviors’ [21], ‘incentive salience’ [22], and simply ‘drive’ [23]. Furthermore, it is clear that these DA systems affect not only reward processing, but a number of related functions, including punishment [24], decision-making [25,26], cognition [27], reward prediction [28,29], and reward valuation [30-32].

**Figure 1:**
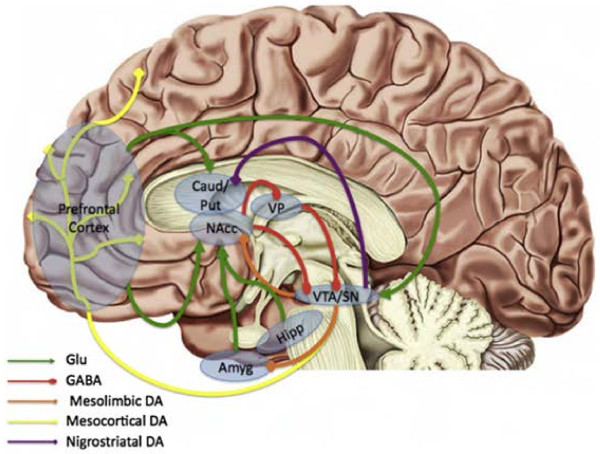
**Schematic illustration of the DA pathways and circuitry that regulate dopamine (DA) release in the human brain.** The DA-containing neurons in the ventral tegmental area (VTA)/substantia nigra (SN) project to the nucleus accumbens (mesolimbic pathway; orange), to the cortex (mesocortical pathway; yellow) and caudate putamen (nigrostriatal pathway; purple). DA neuron firing rates are maintained at tonic levels in part due to steady-state inhibitory firing from the ventral pallidum. Excitatory glutamatergic fibers (green) project from the prefrontal cortex, amygdala, and hippocampus, that synapse on striatal targets, including the nucleus accumbens (NAc). The NAc sends GABAergic projections (red) to the ventral pallidum that suppress ventral pallidum inhibition of the VTA, thereby facilitating phasic burst firing of ventral tegmental area DA neurons. Note: Placement of structures is only approximate. Amyg, amygdala; Caud, caudate; DA, dopamine; GABA, GABAergic projections; Glu, glutamatergic projections; Hipp, hippocampus; Put, putamen; VP, ventral pallidum. (Figure and legend adapted with permission from Treadway and Zald [19].)

#### Organization and criterion for disorders included in this review

This review is organized as follows. First, we briefly outline the neurobiology of the reward system and discuss potential molecular and cellular mechanisms underlying dysregulated reward-pathway functions. Next, animal models of neurodevelopmental and psychiatric disorders that involve dysregulated reward systems are reviewed, followed by a review of clinical studies of reward-circuitry function within multiple disorders, with a particular emphasis on functional neuroimaging studies and molecular-imaging studies that address striatal DA transmission. We first present psychiatric disorders (i.e., substance-use disorders, affective disorders, eating disorders, and obsessive–compulsive disorder (OCD)), then neurodevelopmental disorders (i.e., schizophrenia, attention deficit/hyperactivity disorder (ADHD), autism spectrum disorders (ASDs), Tourette’s syndrome (TS), and conduct disorder/oppositional defiant disorder (CD/ODD)), and finally genetic syndromes (Fragile X syndrome (FXS), Prader–Willi syndrome (PWS), Williams syndrome (WS), Angelman syndrome (AS), and Rett syndrome (RS)). For all disorders, we emphasize how phenotypic expression of disparate symptoms may be interpreted within the context of reward-processing deficits. We also include brief summaries of effective pharmacologic treatments for each disorder affecting DA function. We conclude with suggestions for directions for future research aimed at treatment of reward-system dysfunction. To constrain the scope of this review, we have considered only disorders primarily considered as psychiatric and neurodevelopmental disorders and genetic syndromes. We therefore have not included disorders such as Huntington’s disease and Parkinson’s disease that are both considered to be neurodegenerative diseases coded as Axis III conditions in the DSM (‘general medical conditions’) and that are typically listed as an associated feature of an Axis I condition [1].

Although this review focuses primarily on DA transmission in the mesolimbic pathway, multiple other brain neurotransmitter systems are crucially involved in reward processing. For example, pharmacological studies in rodents indicate that distinct serotonin-receptor subtypes expressed both within and outside the mesolimbic system can modulate responses to either natural rewards or drugs of abuse [33]. Whereas norepinephrine has been traditionally associated with stress responses, both DA and norepinephrine are released in an opposing manner in the bed nucleus of the stria terminalis, in response to either aversive or rewarding taste stimuli, indicating interplay in these chemical systems [34]. Endogenous opioids, including endorphins, enkephalins, and dynorphins, can modulate DA transmission in the mesolimbic pathway [35]. Substance-abuse studies have shown that alcohol, which promotes gamma-aminobutyric acid (GABA)_A_ receptor function, may inhibit GABAergic terminals in the VTA and hence disinhibit these DA neurons, thereby facilitating mesolimbic reward-pathway transmission [36]. Abusive opiates such as heroin function similarly, but in an indirect manner: they inhibit GABAergic interneurons in the VTA, which disinhibits VTA DA neurons and thus enables activation of the reward pathway. These observations highlight the importance of GABA transmission in the VTA for reward processing. Finally, synaptic transmission in the NAc relies on glutamatergic inputs from multiple areas, and glutamate can induce modifications in dendritic morphology, ionotropic glutamate receptors, and the induction of synaptic plasticity in the NAc, implicating glutamatergic transmission in coordinating reward processing [37,38]. These examples indicate that processing of rewarding information involves a complex crosstalk between the DA mesolimbic system and other neurotransmitters, and that interdependency probably occurs across multiple systems and circuits. To simplify this considerable complexity, we aim in this review to summarize the importance of animal models and clinical findings in addressing dysfunction in systems mediating reward processing (broadly defined) by focusing on striatal DA responses to rewarding stimuli.

### Brain reward circuitry

Responses to rewards are mediated primarily by the ascending mesolimbic DA system that is highly similar between humans and other animals (Figure 1 shows structures that will be discussed as part of the mesolimbic DA system) [39]. Although the terms ‘reinforcement,’ and ‘reward’ are often used interchangeably, these terms have discrete behavioral definitions, and describe largely distinct neurobiological processes. Indeed, there are multiple constructs mediated by the mesolimbic system, and at least four such systems have been described in depth in numerous seminal reviews [39-43]: 1) reward motivation, also termed anticipation (typically subsuming what is colloquially described as ‘wanting,’) refers to processes that facilitate anticipation of reward and approach behaviors towards biologically relevant goals, including reward valuation, willingness to expend effort to obtain rewards, reward prediction, and reward-based decision-making [44]; 2) reward outcome (or the hedonic responses widely referred to as ‘liking’ or ‘pleasure’) includes both consummatory behaviors during reward obtainment and the processes associated with regulation of such behaviors [45]; 3) reward learning includes reward processes that shape the experience-dependent learning that guides future behaviors [46]; and 4) reward-related habitual behavior reflects those processes that are initiated based on reward feedback, but that persist even in the absence of such feedback [47,48].

The neurobiological bases of reward-processing behaviors are well understood in animal contexts [41,49-51], and cognitive affective neuroscience techniques have facilitated the investigation of reward circuits in human clinical contexts [52,53]. The mapping of brain-reward regions began with the seminal discovery that animals are willing to work to obtain electrical stimulation to mesolimbic brain regions [54]. Subsequent research showed that activity of DA neurons within mesolimbic pathways that project from the VTA to the NAc serve to reinforce responses to both primary rewards (for example, food) and secondary rewards (for example, money) [55]. Reward information is processed via a limbic cortico-striatal-thalamic circuit that interdigitates with the mesolimbic DA pathway [56,57], and the NAc serves as a DA-gated mediator for information passing from the limbic system to the cortex [58]. This tract is composed of projections from A10 cells in the VTA to cells in limbic areas, including the NAc, the amygdala, the olfactory tubercle, and the septum [59]. This tract has been linked to primary rewards, secondary rewards, and emotional processes, and is part of the limbic-striatal-pallidal circuit that is involved in motivated behavior [60].

Primary DA centers in the mammalian brain are located in two mesencephalon structures: the substantia nigra and VTA. These distinct brain nuclei contain DA-synthesizing neurons that project to the NAc (mesolimbic pathway), the cortex (mesocortical pathway), and the caudate putamen (nigrostriatal pathway). The central node within the mesolimbic DA reward system is the NAc within the ventral striatum. The NAc, along with the extended amygdala, mediates reward-based drive and motivation [61,62], and receives afferents from a number of limbic regions, including the medial and orbital frontal cortices, the hippocampus, and the amygdala [62]. Of particular relevance to reward-based processes is the ventromedial shell of the NAc (the core region regulates cognition and motor control) [63], that serves as an interface between limbic and motor circuits, translating emotions into actions [64]. For this reason, as will be reviewed below, most animal models and clinical neuroimaging studies on reward-related processes focus on functioning of the NAc, and of related afferent and efferent projection regions within the striatum and frontal lobes.

#### Mechanisms of neurotransmission in the mesolimbic reward pathway

**Figure 2:**
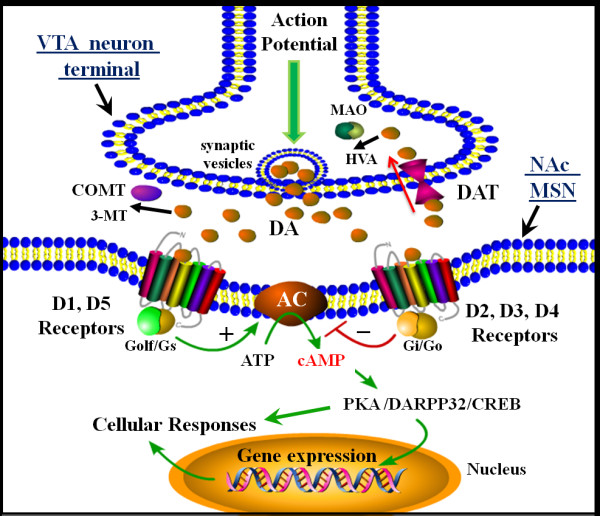
**Schematic illustration of cellular mechanisms of neurotransmission in the mesolimbic dopamine (DA) reward pathway.** Shown is a synapse between a ventral tegmental area DA neuron axon terminal and a medium spiny neuron (MSN) in the nucleus accumbens (NAc) in the ventral striatum. Transmission begins with an action potential that arrives to the terminal, inducing synaptic vesicle fusion and release of DA. The release of DA into the NAc stimulates various populations of MSNs, whose response to the transmitter depends on the types of DA receptors they express. DA stimulation of neurons containing D_1_ or D5 receptors (so-called D_1_-like receptors) results in activation of heterotrimeric Golf/Gs proteins, which activate the enzyme adenylyl cyclase, resulting in the synthesis of the second messenger cAMP. In contrast to this mechanism, DA stimulation of MSNs that express D_2_, D3 or D4 (or D_2_-like receptors) activate sheterotrimeric Gi/Go proteins, which inhibit adenylyl cyclase activity to decrease cAMP. The level of intracellular cAMP controls the activation of protein kinase A, which regulates additional signaling molecules including dopamine- and cAMP-regulated neuronal phosphoprotein of 32 kDa (DARPP-32) and the transcription factor cAMP response element binding (CREB) protein, both of which can modulate gene expression and additional cellular responses. The response to DA is generally terminated when DA is removed from the synapse by reuptake via the DA transporter (DAT). After reuptake, the transmitter can be repackaged into synaptic vesicles or may be degraded by the enzyme monoamine oxidase, resulting in the DA metabolite homovanillic acid. In addition, the enzyme catechol-*o*-methyltransferase (COMT) may also control DA levels by breaking down DA to 3-methoxytyramine (3-MT), AC, adenylyl cyclase; ATP; adensosine triphosphate; cAMP; cyclic adenosine monophosphate; HVA, homovanillic acid; MAO, monoamine oxidase; VTA, ventral tegmental area.

The molecular and cellular mechanisms that facilitate neurotransmission in the mesolimbic DA reward pathway involve the cellular elements modulating synaptic DA neurotransmission, including neurotransmitters, transporters, receptors, G proteins, second-messenger-generating enzymes, ion channels, and immediate early response genes that regulate neuronal functions (Figure 2) [65-67]. Afferents from the VTA of the mesolimbic DA system project outward, and primarily terminate onto the MSNs, which are the principal cell type in the NAc, and produce and secrete GABA, the main inhibitory neurotransmitter used in the CNS. These MSNs are also the main projection or output neurons of the NAc.

Neurotransmission within the mesolimbic pathway begins with an action potential that is generated in VTA neurons, resulting in the presynaptic release of DA. Neurotransmission of the DA signal to MSNs in the NAc is mediated by binding to specific DA receptors. These DA receptors are part of the Gprotein-coupled receptor superfamily, and upon binding DA, activate heterotrimeric G proteins (Golf/Gs or Gi/o) that in turn regulate the activity of effector proteins such as ion channels, or the enzyme adenylyl cyclase that produces the second messenger cAMP [65]. Five distinct DA receptors (D_1_ to D_5_) can mediate neurotransmission, and are coupled positively to activation of adenylyl cyclase (D_1_ and D_5_ receptors) or negatively to inhibition of adenylyl cyclase (D_2_, D_3_, D_4_). Consequently, MSNs that express D_1_-like receptors become activated by DA, resulting in an increase in cAMP synthesis, whereas MSNs that express D_2_-like receptors respond to DA by decreasing cAMP synthesis. cAMP in turn activates protein kinase A, that phosphorylates target proteins resulting in modulation of neuronal activity, gene expression, and target-protein functions. The response to DA in this neuronal pathway is terminated by reuptake of DA into the presynaptic neuron terminals, which is controlled by the DA transporter (DAT). In addition, the enzymes monoamine oxidase (MAO) and catechol-*o*-methyltransferase (COMT) can regulate DA levels by breaking down DA to the metabolites homovanillic acid or 3-methoxytyramine (3-MT), respectively.

Given its anatomical organization, the NAc is considered a limbic–motor interface [68] translating information about rewards into appropriate behavioral responses to obtain these rewards. The major effect of DA transmission is to modulate the sensitivity of NAc MSNs to other types of input. For example, DA modulates the sensitivity of MSNs to excitatory glutamatergic projections from pre-frontal and limbic regions, and thereby modulates firing activity of NAc neurons [35,69]. The result of DA transmission on NAc neuronal firing is largely determined by the types of DA receptors expressed in post-synaptic MSNs. Although the precise causal link between DA release and NAc cell firing is unclear, D_1_ and D_2_ receptors are generally considered to exert opposite effects at the cellular level, with D_1_-like receptor-expressing cells responding to DA with excitatory increases in firing activity, and D_2_-like receptor-expressing cells responding with decreased firing activity. However, in the context of DA release in the brain, a cooperative interplay between NAc neurons that encode reward information probably occurs. For example, DA increases spike firing in MSNs, requiring coactivation of both D_1_ and D_2_ receptors [70]. Furthermore, transmission of DA to the NAc occurs with the same temporal resolution as NAc neuron-patterned cell firing, and this DA release and firing are coincident during goal-directed actions in rodents [71]. In addition, the frequency of firing activity of VTA neurons may be a key component in modulating the mesolimbic reward pathway and encoding reward information. Studies using channel rhodopsin to precisely control VTA neuron firing activity suggest that phasic, but not tonic, activation of VTA neurons is sufficient to drive behavioral conditioning to rewards and elicit DA transients [72], and thus indicates the likely importance of the frequency of VTA neuron firing activity.

#### Potential molecular and cellular mechanisms underlying dysregulated reward systems

Disruption of molecular, cellular, or circuitry mechanisms that are essential for the reward system may, in theory, result in aberrant reward-system function. Although a primary (or even common) molecular mechanism for dysregulating the reward system has yet to be identified, we briefly consider in the following section some of the potential molecules and mechanisms that may underlie abnormal reward processing.

Because the major neurotransmitter mediating mesolimbic transmission is DA, alterations in the synthesis, release, or reuptake of DA may result in an abnormally functioning reward system. Amphetamines and cocaine mediate their effects in the mesolimbic pathway by increasing the release of DA. Cocaine and amphetamines, both of which directly interact with the DAT, exert their effects, at least in part, by blocking (in the case of cocaine) or reversing the direction of (in the case of amphetamine) this transporter, resulting in increased synaptic DA [73]. Indeed, chronic administration of cocaine upregulates striatal DAT expression in rhesus monkeys, an effect that persists for more than 30 days after cocaine withdrawal [74]. Increased DA-transporter expression has also been shown in post-mortem analyses of brain tissue from human subjects addicted to cocaine [75]. Such studies indicate that alterations in DAT expression or function can result in an altered reward system in response to drugs of abuse.

Similarly, alteration in the expression or regulation of DA receptors would also be expected to dysregulate reward-system functions. Altered DA receptor function could involve increased or decreased receptor expression or signaling responsiveness to DA thereby altering the reward system. For example, the DA hypothesis of schizophrenia suggests that excess mesolimbic DA levels may be pro-psychotic, and involve alterations in the activity of striatal D_2_ receptors, which are the major site of action for typical antipsychotic medications [76]. There is clear evidence of dysregulated striatal DA function in schizophrenia [77], and a meta-analysis of multiple studies indicated a significant increase in striatal D_2_ receptors in patients with schizophrenia who were not on medication [78]. Studies have also suggested an increased affinity of D_2_ receptors for DA in schizophrenia, which may produce D_2_ receptor supersensitivity in the NAc, contributing to psychosis [79]. In an interesting animal model correlate to these studies, transient overexpression of D_2_ receptors in the striatum of mice resulted in deficits in prefrontal working memory, resembling some of the features of human schizophrenia [80]. Studies such as these indicate that alterations in DA receptor expression (or function) can result in a dysfunctional reward system.

Molecules that are activated downstream of DA receptor signaling in the NAc also play important roles in mediating reward responses and changes in their function may also dysregulate the reward system. These molecules include the heterotrimeric G proteins activated by DA receptors and also the adenylyl cyclases. Interestingly, genetic knockout of adenylyl cyclase type 5 in mice prevents the reward response to opioids such as morphine [81]. Further down in the DA signaling pathway of MSNs is the DA- and cAMP-regulated phosphoprotein of 32 kDa (DARPP-32) (Figure 2). DARPP-32 is activated by D_1_ receptor cAMP signaling in the NAc by protein kinase A phosphorylation, that regulates the activity of protein phosphatase (PP)-1 [82]. Phosphorylated DARPP-32, by inhibiting PP-1, acts in a combined manner with other protein kinases to increase the level of phosphorylation of various downstream effector proteins, and modulation of protein phosphorylation by DA is thought to play an important role in drug reward. DARPP-32 may thereby influence the long-term neuronal adaptations associated with natural rewards or with rewards from drugs of abuse [83,84]. Support for this concept is provided in genetic models in mice lacking the DARPP-32 gene, which results in decreased responses to cocaine in conditioned place preference behaviors [85]. Therefore, alterations in DARPP-32, PP-1, and the phosphoproteins that these regulate in MSNs, may dysregulate the reward pathway.

Two transcription factors, ΔFosB and cAMP response element binding protein (CREB), are activated by DA receptor signaling in the NAc, and both are important mediators of reward responses because they control the expression of numerous genes. One of the most dramatic examples of protein expression induction is in the transcription factor ΔFosB, a Fos family protein, which accumulates in the NAc after chronic exposure to drugs of abuse, including alcohol, amphetamine, cannabinoids, cocaine, nicotine, opiates, and phencyclidine [86,87]. Overexpression of ΔFosB in the NAc increases behavioral responses to cocaine, opiates, sucrose and wheel-running, including increased incentive drive for these rewards. Conversely, blockade of ΔFosB function in the NAc by overexpression of a dominant negative antagonist causes the opposite effects [88].

CREB is another transcription factor that is directly activated by protein kinase A in response to DA signaling in the NAc. Activation of CREB seems to produce similar behavioral responses to rewarding stimuli: in numerous experimental systems, increased CREB activity in the NAc is negatively related to behavioral responses to cocaine, opiates, and alcohol [86,88-90]. CREB is also induced in the NAc by natural rewards (such as sucrose), and similarly reduces an animal's sensitivity to the rewarding effects of sucrose [89]. Therefore, any changes in the activation and induction of CREB, ΔFosB, (and probably many other transcription factors) would be expected to regulate or dysregulate the reward system.

Finally, although the molecules highlighted here are clearly involved in DA mesolimbic transmission and reward responses, this represents only a brief overview and readers are encouraged to see other recent reviews of this topic [86,91-93].

### Considerations for animal models that focus on reward-system function

Animal models, particularly those using rodents, have provided key mechanistic insights that have elucidated the neurobiology of the brain reward system. Although animal models cannot recapitulate the entire spectrum of phenotypes apparent in clinical presentations of illness, they provide powerful approaches for experimental studies using various environmental, genetic, pharmacological, and biological manipulations. With regard to studying behavior, a high degree of experimental control can be achieved by precisely controlling the animal's life experiences, environment, diet, and history of drug exposure, enabling inferences to be made concerning the causality of effects seen in experimental studies. However, for complex psychiatric disorders with largely unknown genetic etiologies, environmental insults, specific pathologies, or biomarkers, the building of animal models with high construct validity has not yet been possible [94]. With this limitation in mind, an alternative strategy has been to develop mouse genetic models (for example, knockout or transgenic mice) of psychiatric disorders with relevant behavioral phenotypes (face validity) that are responsive to pharmacotherapies that are clinically effective (predictive validity).

### Considerations for clinical studies that focus on reward-system function

Primary rewards are vital to gene propagation, and thus responses to such stimuli have been shaped by evolution to elicit approach-oriented behaviors. These stimuli include food and sexual behavior (given that sustenance and procreation are crucial for the survival of a species [95,96]), and social interactions with conspecifics [39,97]. Nonclinical human neuroimaging studies indicate that the mesolimbic DA response to primary rewards may operate similarly in humans in response to more abstract, or secondary, rewards such as monetary incentives [98-100]. Recent evidence suggests a common ‘neural currency’ for coding monetary and primary (for example, food) rewards [101]. Thus, most clinical studies investigating responses to rewards have used monetary incentives as a proxy for primary rewards, because money is adaptable to the research environment, may be parametrically scaled, may be won or lost, and may be delivered at precise intervals.

It should be noted that few of the preclinical and clinical studies reviewed here involve longitudinal data collection, and it is difficult to make any inferences about the developmental nature of reward-processing systems in the disorders reviewed. In this regard, although our goal is to propose a possible common framework for conceptualizing a range of seemingly disparate phenotypes and possibly to ultimately identify novel biological markers and influence nosological classification, inferences about etiology must be appropriately cautious in the context of largely cross-sectional data.

### Psychiatric disorders

#### Substance-use disorders

Perhaps the greatest convergence of empirical evidence supporting reward-network dysfunction in psychiatry emanates from research on substance-use disorders [102]. The 12-month prevalence estimates for substance-use and abuse disorder are about 3.8% [103]. Contemporary theories addressing the pathophysiology of substance-use disorders highlight altered motivational states, cognitive control, inhibitory function, and decision-making, mediated in large part by dysfunctional output of mesolimbic and mesocortical brain systems [104-107]. Although the scope of this review is constrained to a consideration of reward processes, rather than to related constructs such as inhibition and impulsivity, it should be noted that the ‘impulsivity hypothesis’ of addiction vulnerability stresses shared neurobiology and patterns of heritance between risk for addiction disorders and conduct disorder [108], including evidence of intergenerational transmission of both alcoholism risk and impulsivity in large-scale twin studies [109], and common patterns of enhanced behavioral sensitivities to reward stimuli [110] and risky decisions [111,112].

The rewarding effects of drugs of abuse derive in large part from the sizeable increases in extracellular DA in limbic regions, and in the NAc in particular, during drug use [113,114]. In addition, drug-induced increases in striatal DA have been linked with subjective feelings of euphoria [115,116]. The firing of DA cells that accompanies drug use encodes a number of reward properties, including reward expectancy [117], reward learning [118], and the consolidation of contextual memories [119]. All of these processes are believed to contribute to the intense motivation to attain drugs of abuse [120].

It has been proposed that the crucial mechanism for the development of addiction is drug-induced activation of DA transmission in the mesolimbic pathway, also referred to as the ‘dopamine hypothesis of addiction’ [121-123]. To better understand the neurobiology of drug abuse and addiction in humans, several animal models have been developed to investigate different aspects of drug addiction [122,124]. Among these, the models that incorporate self-administration of drugs are thought to best capture the human condition because animals voluntarily seek drugs and because drugs that are self-administered by animals correspond well with those that have abuse potential in humans.

##### Preclinical models

From mechanistic neurobiological and behavioral studies in rodents, it has become clear that the mesolimbic pathway is a key component for the rewarding effect of drugs of abuse, and is essential for behaviors related to drug reward, salience, and motivation [122]. For example, using rodent models, researchers have determined that nearly all psychoactive drugs of abuse (for example, cocaine, amphetamines, alcohol, opiates, cannabinoids, nicotine) induce alterations in the transmission of DA within the mesolimbic pathway, with most of these drugs increasing extracellular concentrations of DA [122]. Studies using an *in vivo* microdialysis technique, which measures minute changes in brain neurotransmitter levels in the behaving animal, have shown that drugs of abuse can increase tonic DA concentrations in the NAc. In addition, studies using fast-scanning cyclic voltammetry, which can detect the level of DA release in the intact brain on a timescale of seconds, have shown an increased frequency of spontaneous phasic DA signals in the NAc in response to cannabinoids and nicotine in awake, behaving animals [125,126], and also temporally distinct DA signals in response to cocaine [127].

Dopamine neurotransmission is strongly implicated in the reinforcement of self-administering drugs or electrical stimulation in animals. The seminal animal research by Olds and Milner [54] provided the initial foundation for our modern understanding of brain-reward mechanisms. In those pioneering studies, rats were given the ability to self-administer electrical stimulation to various brain regions including the mesolimbic pathway. The rats persistently and repeatedly chose to stimulate the VTA mesolimbic DA pathway (but not other brain areas), often to the exclusion of other behaviors. Behavioral studies in rodents also indicate that DA is essential for the self-administration of drugs of abuse for which the mesolimbic pathway has been identified as a crucial substrate [114,128]. Drug self-administration is the ‘gold standard’ of animal models of drug abuse [122,129]. In the typical drug self-administration procedure, animals obtain a drug by performing a simple behavior (such as pressing a lever), and animals will readily self-administer the same drugs that are abused by humans [130].

The importance of mesolimbic DA transmission to drug self-administration is supported by pharmacological and lesion studies. Direct DA receptor agonists can mimic the effects of substances of abuse, and these agonists are self-administered both systemically and locally into the NAc in rats and monkeys [131-133]. By contrast, DA receptor antagonists administered systemically increase the rate of operant responding for cocaine in animals [134-136]. In addition, lesion or inactivation of the mesolimbic DA system in the VTA [137,138] or in the NAc [139-143] decreases cocaine, amphetamine, heroin, and nicotine self-administration in rats. These findings indicate the crucial importance of the mesolimbic DA system in drug-taking.

##### Clinical studies

There is a confluence of clinical evidence that substance-use disorders are characterized by relative hyperactivation of mesolimbic regions in response to drug cues (that is, increased reward motivation). This pattern is evident across various subtypes of substance-abuse disorders, suggesting the central involvement of striatal regions encoding reward prediction and/or anticipation in substance-abuse disorders. Wexler and colleagues [144] presented cocaine-addicted subjects with videotapes containing cocaine-associated cues, and reported relatively increased anterior cingulate cortex (ACC) activation during the presentation of the cocaine cues, despite decreased overall frontal lobe activation. Further, these effects were evident even in the absence of self-reported cravings, suggesting that brain-imaging effects did not simply recapitulate experiential responses to the cocaine cues “Buhler and colleagues” [145] assessed anticipatory responses to cues predicting cigarette and monetary rewards in nicotine-dependent smokers and non-dependent occasional smokers. The non-dependent group showed relatively increased mesocorticolimbic reactivity to stimuli predicting monetary reward compared with stimuli predicting cigarette rewards, and subsequently spent relatively more effort to obtain money relative to cigarettes. By contrast, the nicotine-dependent group showed equivalent responses to both categories of reward cues, and anticipatory mesocorticolimbic activation predicted subsequent motivation to obtain both rewards, suggesting an imbalance in reward motivation in response to drug-predicting cues relative to monetary cues in those with nicotine dependence. Myrick and colleagues [146] reported that activation in the NAc, anterior cingulate, and left orbitofrontal cortex in response to alcohol images predicted cravings in alcoholics. Oberlin and colleagues [147] reported that the magnitude of striatal activation to alcohol cues (the odors of the preferred alcohol drink) in heavy drinkers was modulated by antisocial trait density. Finally, Filbey and colleagues [148] showed that regular marijuana users who abstained from use for 72 hours were characterized by relatively increased reward-circuitry activity, including the VTA, thalamus, ACC, insula, and amygdala, in response to tactile marijuana cues. These studies reflect the overall pattern of data in a range of substance-abuse disorders, which shows relatively increased mesolimbic activation in response to drug cues, accompanied by increased states of reward motivation in response to these cues [148].

In contrast to the hyperactive responses of reward circuitry to drug-related cues, there is evidence that substance-use disorders are alternatively characterized by a reduced motivation for non-drug rewards [106]. As a number of researchers have described [106,149], substance-use disorders are typically accompanied by decreased reward motivation for typical and non-pathological rewards, a phenomenon that has been variously termed ‘motivational toxicity’ [150] and ‘reward-deficiency syndrome’ [151]. For example, Asensio and collegues [152] reported hypoactivation of the dorsal and ventral striatum and the dorsomedial pre-frontal cortex when cocaine addicts viewed pleasant images not linked to substance cues. Gilman and Hommer [153] reported subjective hypoarousal to normative positive images in alcohol-dependent participants. Bühler and collegues [154] reported mesocorticolimbic hypoactivation during monetary-reward motivation in nicotine-dependent participants, which predicted motivation to obtain rewards. Andrews and colleagues [155] reported decreased NAc activation to monetary-reward outcome that predicted family history of alcoholism. In a study using multi-modal psychophysiological measurements, Lubman and colleagues [156] reported decreased arousal ratings and physiological measures of reward motivation to pleasant pictures relative to drug-cue images in opiate-dependent participants. Luo and colleagues [157] found relatively decreased right ventral striatal activation during the anticipation of delayed relative to immediate monetary rewards in cigarette smokers (that is, decreased reward motivation for delayed monetary rewards). However, Jia *et al*. [158] reported that treatment-seeking adults with cocaine dependence were characterized by striatal hyperactivation during monetary-reward outcome and that striatal activation during reward motivation predicted treatment outcome.

Attenuated motivation for non-drug rewards has also been reported in younger populations at risk for substance abuse. Schneider and colleagues [159] found that adolescents with risky substance- use patterns had reduced striatal activity relative to low-risk adolescents during monetary-reward motivation [17][ Similarly, Peters and colleagues [160] reported reduced ventral striatal responses during the anticipation of food reward in adolescent smokers. Notably, Andrews and colleagues [155] found this effect in family members of those with substance abuse, suggesting that this pattern may be evident even in the absence of the direct effects of repeated drug use on the brain. Overall, these studies highlight that the effects of altered mesolimbic function in substance-use disorders may be characterized not only by increased reward motivation for substance-related stimuli, but also by decreased reward motivation for natural rewards (but there are exceptions [161]), which may lead to increased drug-seeking behaviors. In this regard, Koob and Le Moal [162] described an allostatic mechanism through which the reward system may become desensitized with repeated exposure to addictive drugs, due to gradual modulation of an organism’s ‘set point’ of responsivity to external rewards.

Molecular-imaging studies of substance-use disorders have focused on imaging the D_2_ post-synaptic receptor [106,163]. There are multiple lines of evidence that cocaine dependence is associated with a decrease in D_2_ receptor binding [164-167], a pattern that seems to persist after disease remission [165]. Decreases in D_2_ receptor binding have also been found in heroin addiction [168], alcohol dependence [169,170], methamphetamine abuse [171,172], prompting a number of researchers to posit that low D_2_ receptor availability may serve as a biomarker for substance abuse, potentially reflecting an altered sensitivity to various rewards [173-175]. Although these molecular-imaging studies suggest decreased reward motivation in addiction that is consistent with the ‘reward-deficiency syndrome’ hypothesis of addiction, functional brain-activation studies paint a less consistent picture, probably due to variability in samples, task demands, patient characteristics, and unknown effects of a history of addictive behavior on functional responses to reward stimuli. Future research that combines molecular and functional imaging approaches will be necessary to elucidate the causes and consequences of altered reward processing in substance-use disorders in at-risk individuals [176].

##### Dopaminergic treatments

A number of agents that modulate functional output of DA systems are effective first-line treatments for substance-use disorders [177]. Modafinil is a non-amphetamine stimulant with DA and glutamatergic effects, and with moderate effectiveness for the treatment of cocaine dependence [178] and possibly methamphetamine dependence [179]. Bupropion is a DA and norepinephrine reuptake inhibitor that is an effective treatment to promote smoking cessation [180]. Dextroamphetamine causes release of DA (as well as norepinephrine and serotonin) and is an effective treatment for amphetamine abuse [181]. Finally, risperidone, a D_2_-receptor antagonist, has shown promise for the treatment of methamphetamine abuse [182], and aripiprizole, a partial D_2_ agonist is a promising treatment for amphetamine abuse [183].

#### Affective disorders

Unipolar major depressive disorder (MDD) is associated with significant psychosocial and medical morbidity and mortality [184-186], and has an estimated lifetime prevalence of 14.6% [187]. Anhedonia, the decreased response to pleasurable stimuli, is a defining symptom of the disorder to the extent that MDD may be diagnosed even in the absence of depressed mood if anhedonia and other secondary symptoms are present [1]. Anhedonia is also a central feature of a number of neurobiological theories of depression that posit that deficits in emotional and motivational responses to appetitive stimuli are core features of the disorder [188], and the anhedonic endophenotype of MDD is perhaps the most well supported [10].

##### Preclinical models

Because anhedonia is a defining symptom of affective disorders, animal models of hedonic deficits have been addressed in preclinical models of affective disorders. Chronic mild stress has been reported to induce an anhedonic-like state in rodents,that resembles the affective disorder phenotypes in humans [189]. In particular, Willner and colleagues originally reported that chronic and sequential exposure of rats or mice to a mild stress regimen caused decreases in responsiveness to rewards [190,191], commonly reported as a decrease in the consumption of and preference for sucrose solutions, and a decrease in the rewarding properties of pharmacological and natural rewards in the place preference behavioral paradigm [189,192-194]. The chronic stress paradigm is considered to have a greater etiological relevance and face validity in mimicking MDD than other animal models, and therefore has become one of the most widely used preclinical paradigms of affective disorders [195]. Chronic mild stress causes significant reductions in absolute and relative sucrose intake in rats, that is associated with a decrease in striatal DA activity, and is reversed after chronic antidepressant administration with imipramine [196]. Decreased DA release to the NAc has been shown to occur after exposure to chronic repeated or an unavoidable stress regimen in rats [189,197,198], suggesting that stress significantly reduces mesolimbic DA transmission in rodent models. Altered DA function may also be related to changes in D_1_ receptors, which have been shown to alter functional output in the rat limbic system after chronic unpredictable stress [199]. Therefore, stress-induced neurochemical changes, including decreased DA activity in the mesolimbic pathway, contributes to decreased natural reward (sucrose)-seeking in this animal model of affective disorders.

##### Clinical studies: unipolar major depressive disorder

Reward-system dysfunction in MDD is well established [200-202]. Behavioral studies have reliably found that individuals with MDD show a blunted response to a range of rewarding stimuli [203-205]. Reward learning has also been found to be impaired in MDD [206], and this impairment is correlated with the severity of anhedonic symptoms [207]. Additionally, the severity of MDD has been found to correlate strongly with the magnitude of the rewarding effects of administration of oral D-amphetamine, which increases DA availability [208], and anhedonic symptoms in the general population predict rewarded effort-based decision-making [209].

Functional neuroimaging studies in MDD have consistently indicated hypoactivation in reward-processing regions, including the dorsal and ventral striatum [210-214] and a host of other reward structures, including the medial prefrontal cortex [215,216], the pregenual and subgenual anterior cingulate, and the medial frontal gyrus [217,218].

Reduced mesolimbic activity in MDD has been found during reward anticipation and outcomes in both adults and children [210,219-227] and during reward learning [206]. For example, Smoski *et al*. [228] reported that during a gambling task, outpatients with unipolar MDD had reduced striatal activation during reward selection, reward anticipation, and reward feedback (but see Knutson *et al*. [229] for a report of intact striatal function but increased ACC activation in depression during reward anticipation). In a follow-up study, Dichter and colleagues [230] reported that when these same patients were treated with behavior-oriented psychotherapy designed to increase interactions with potentially rewarding situations, striatal regions showed increased functioning during reward anticipation, similar to results of Forbes *et al*. [231]. Finally, there is also evidence that reward-network function shows greater impairment in MDD while patients are processing pleasant images relative to monetary rewards [232].

Altered reward-network responsivity may also be characteristic of individuals with a history of MDD but without significant current symptoms, suggesting that anhedonia may represent a trait marker of MDD vulnerability, independent of current MDD state [233,234]. McCabe *et al*. [235] found decreased ventral striatal activation during reward outcome in response to the sight and flavor of chocolate in euthymic individuals with a history of depression, and Dichter and colleagues [236] reported reward-network hyperactivation during reward anticipation and hypoactivation during reward outcomes in individuals with remitted unipolar MDD. Although studying patients with remitted depression is not sufficient to establish reward-processing deficit as a trait marker of depression, given that the effect of past illness and treatments on brain function may not be conclusively excluded, it is nevertheless a necessary initial step to identify this disease trait. It also has the advantage of mitigating the potential confounding effects of current mood state, illness severity, non-specific effects of chronic illness and stress, and effects of psychotropic medication usage [237,238]. Thus, examining linkages between brain function and a history of MDD holds the promise of ultimately aiding in the identification of trait-like endophenotypic vulnerability markers predictive of MDD onset before clinically impairing symptoms appear.

Further converging evidence of the crucial role that reward-network functioning plays in MDD is found in literature documenting the remarkable consistency with which antidepressant response is predicted by pretreatment functioning of the ACC. The ACC plays a central role in processing positively valenced emotions [239] and other rewards [240], and in coding value representations of anticipated rewards [241], as shown in studies of sleep deprivation [242-245], psychopharmacological intervention [246-250], cognitive behavioral therapy [251,252] and a combined approach of therapy and psychopharmacological intervention [231].

Given the linkages between anhedonia, unipolar MDD, and mesolimbic dysfunction, and the prevalence of anhedonia in a number of other Axis I disorders, including bipolar disorder, schizophrenia, and post-traumatic stress disorder, an area of neglected study is the direct comparison between MDD and these other conditions. A notable exception is a study by Lawrence *et al*. [253], in which euthymic and depressed patients with bipolar disorder and patients with unipolar depression viewed faces with varying emotional intensities. Whereas the bipolar group was characterized by differential ventral striatal responses to nearly all emotion categories, the unipolar group was characterized by blunted response to happy but not sad stimuli, suggesting that diminished reward outcome to pleasant stimuli may uniquely characterize unipolar MDD relative to bipolar MDD. Future three-group studies comparing MDD with other disorders characterized by anhedonia are needed to distinguish similarities and differences between these conditions with respect to processing reward stimuli.

Molecular-imaging studies of unipolar depression have reported decreased monoamine signaling, which is consistent with functional brain-imaging data suggestive of altered reward processing [254]. In addition to a substantial body of literature on positron emission tomography (PET) addressing serotonin (5-HT)_2_ receptor density in depression [255,256], DAT-binding potential has received considerable attention. Dunlop and Nemeroff [200] summarized the literature to date addressing molecular-imaging studies of DA signaling in MDD. These studies have indicated increased D_2_ receptor binding in the basal ganglia [257], striatum [258,259], and putamen [260], whereas other studies have reported lower [261] or no difference [259,262,263] in striatal D_2_ transporter binding potential.

##### Clinical studies: bipolar disorder

Bipolar disorder is a mood disorder characterized by one or more episodes of mania, defined as abnormally increased energy levels, cognition, and mood [1], and has an estimated lifetime prevalence estimate of around 1% [264]. Mania has been conceptualized as a tendency to show heightened response to positive emotions and rewards [265], along with excessive goal pursuit and unrealistically high expectancy of success. It has been suggested that these symptoms may reflect upregulation of the mesolimbic DA system in bipolar disorder [266]. Behavioral studies of response to rewards in bipolar disorder indicate deficits in behavioral adaptation to changing reward contingencies [267] and prolonged elevation of mood in response to monetary reward in euthymic patients with bipolar disorder [268]. Reward motivation is also atypical in individuals with bipolar disorder, as shown by a self-report measure of reward responsivity [269] and in eye-tracking studies of monetary gains and losses [270].

Although functional MRI studies have identified prefrontal dysfunction in bipolar disorder and manic psychosis, evidence for abnormalities in reward-related neural network function in mania is scarce [271-275]. Although several studies have suggested alterations in the shape [276], size [277,278] and function [274] of the basal ganglia in bipolar disorder, there are only three published functional neuroimaging research studies addressing responses to rewards in bipolar disorder. Abler *et al*. [279] reported decreased NAc activation during monetary-reward outcome, a pattern that was not evident in a group of patients with schizophrenia scanned using the same paradigm. Lawrence and colleagues [253] reported increased ventral striatal and ventral prefrontal cortical responses to mildly happy facial expressions in bipolar disorder. Finally, Jogia and colleagues [280] reported relative ACC hyperactivation during reward processing in bipolar disorder. The paucity of functional brain-imaging research on reward processing in bipolar disorder is striking, given the increasing recognition of reward-system dysfunction in the related conditions of unipolar MDD and schizophrenia, and the conceptual linkages between the symptoms of mania and functions of striatal DA that have been suggested for nearly 20 years [281].

Molecular-imaging studies of striatal DAT availability in bipolar disorder generally suggest increased functional DA throughput (but Suhara *et al*. reported an exception [282]). Amsterdam and Newberg [283] reported higher striatal DAT binding in the right posterior putamen and left caudate in a small number of patients with bipolar disorder; Chang and colleagues [284] reported that unmedicated euthymic subjects with bipolar disorder had significantly relatively higher whole striatal DAT binding; and Anand and colleagues [285] reported relatively lower DAT availability in the dorsal caudate nucleus (DCN) bilaterally. There is also evidence that the presence of psychosis may moderate patterns of DA receptor binding. Specifically, striatal D_2_ receptor signaling seems to be greater in psychotic patients with bipolar disorder [286,287], whereas no differences in D_2_ availability were found between non-psychotic patients with bipolar disorder and controls [288,289].

##### Dopaminergic treatments

Bupropion, a DA and norepinephrine reuptake inhibitor, is an effective antidepressant [290] that seems to specifically increase feelings of positive affect [291]. Other examples of DA agents effective in the treatment of MDD include the selective D_2_/D_3_ receptor agonists pramipexole [292] and piribedil [293] the catechol-O-methyltransferase inhibitor tolcapone, [294], and the preferential presynaptic DA antagonist amisulpride, [295]. Particularly relevant in the present context are previous reports [290,296,297] that although both DA and non-DA agents can be used to effectively treat mood disorders, DA agents generally have superior effects on symptoms of anhedonia, specifically when compared with non-DA agents [19,298-300]. Tremblay and colleagues [226] reported that depressed patients had relatively greater increases in striatal and orbitofrontal cortex activation in response to emotional pictures after administration of dextroamphetamine (a stimulant associated with increased DA release). This highlights the crucial role that the selection of reward-relevant outcome measures will have for studies addressing the efficacy of DA agents in the treatment of mood disorders.

#### Eating disorders Preclinical models

##### Eating disorders Preclinical models

Feeding is a complex process that involves a sensory response to the sight and smell of food, previous feeding experiences, satiety signals elicited by ingestion, and hormonal signals related to energy balance. DA release in specific brain regions is associated with pleasurable and rewarding events, and the mesolimbic system is thought to reward positive aspects of feeding. Some of the most elegant and informative studies clarifying the involvement of DA in feeding and other neurobiological functions come from the studies of Palmiter and colleagues. Zhou and Palmiter [301] developed a DA-deficient mouse by genetically deleting tyrosine hydroxylase, the key enzyme required for the synthesis of L-3,4-dihydroxyphenylalanine (L-DOPA), the chemical precursor of catecholamines. These DA-deficient mice cannot make DA, and are born normal but fail to thrive, have decreased food intake, gradually become hypoactive and hypophagic, and die at 3 weeks of age [301]. However, intervention and treatment of the mice with L-DOPA to restore striatal DA levels to 10% of the levels in normal mice is sufficient to elicit normal feeding behavior and animal survival [302]. Moreover, restoration of tyrosine hydroxylase gene expression using gene therapy was able rescue the deficient feeding behavior in these DA-deficient mice [303]. Using gene therapy to enable DA production within only the caudate putamen restored mouse feeding on regular chow diet, and also normal nest-building behavior, whereas restoration of DA production into the NAc only restored the exploratory behavior [304].

A salient result from these animal studies is that DA transduction in the central or lateral regions of the caudate putamen was sufficient to permanently rescue mice from the starvation that would occur inevitably without daily L-DOPA injections. However, restoration of DA into the NAc in these studies was not sufficient to rescue normal feeding behavior, but this may have been due to an inability to anatomically restore gene expression throughout the entire NAc [304]. Interestingly, when the DA-deficient mice are crossed with obese leptin (Ob/Ob)-deficient mice, the lack of DA blocked the increased feeding behavior normally present in the leptin (Ob/Ob)-deficient mice [305]. Taken together, the DA-deficient mouse studies indicate the essential requirement of DA for normal feeding behavior and survival.

In addition, there is extensive experimental evidence in animal contexts supporting a role for the mesolimbic reward pathway on appetitive and motivational behaviors [306,307]. Mesolimboic DA release is associated with most pleasurable or rewarding events, and food is one type of reward that is often used during the training of animals. There is an increase in DA release (measured in awake, behaving animals by microdialysis or by fastscanning cyclic voltammetry) in the NAc in response to unexpected food rewards or stimuli that predict food rewards [72,308-310]. Moreover, drugs that enhance operant responding for such food rewards, such as amphetamine, are most effective when administered into the NAc, whereas DA receptor antagonists administered into the NAc block the stimulant effects [57,311]. Pharmacological control of the output from the NAc shell can also have profound effects on food consumption [312,313], as does surgical or chemical lesion of the nigrostriatal or mesolimbic DA pathways. These results suggest that DA release in the striatum is required to integrate relevant signals for sustained feeding [301,314,315]. These studies emphasize the importance of DA transmission and the mesolimbic reward pathway for food consumption, feeding behavior, and food rewards in animal models.

##### Bulimia nervosa

Bulimia nervosa (BN) is an eating disorder characterized by recurrent binge eating followed by compensatory behaviors. It typically has its onset during adolescence, has an estimated prevalence of 1-2%, is more common in females, and is characterized by, among other features, impulse-control dysregulation [1,316]. There is high comorbidity between BN and substance abuse, and there is a considerable body of data suggesting that disturbed appetitive behaviors for food in BN may reflect a dysregulation of reward mechanisms that is common to both BN and substance-abuse disorders [317]. Indeed, early hallmark preclinical studies by Hoebel and colleagues [318] highlighted commonalities between BN and addiction disorders in terms of neurobiology, psychopharmacology, neurochemistry, and behavior [319]. Binge eating has also been suggested to serve an emotion regulatory function, and thus has many qualities of reward-mediated behaviors [320].

##### Clinical studies

There has been a small handful of functional neuroimaging studies of response to rewards in BN, with a wide range of rewarding stimuli presented. It is important to note that functional brain imaging studies in eating disorders have the methodological challenge of confounds associated with nutritional imbalances in affected individuals. One way to overcome this is to focus on individuals who are recovered from these disorders at the time of scanning, but it is important to note that such an approach may minimize the extent of brain responsivity differences that would characterize individuals meeting current criteria for this disorder.

Several studies have reported reduced reward motivation for food rewards in eating disorders. Joos and colleagues [321] found reduced activation of the ACC in individuals with concurrent BN during the presentation of visual food cues, and Bohon and Stice [322] reported trends towards decreased right insular cortex activation to the anticipated receipt of chocolate milkshake solution and in the right posterior and dorsal insula in response to milkshake consumption in women with BN. Other studies have found atypical responses during reward outcome for monetary and food rewards. Wagner and colleagues [323] reported that women who had recovered from BN had equivalent DCN responses to monetary-reward outcomes, whereas CN responses in the control group were specifically linked to monetary gains relative to losses. Frank and colleagues [324] reported decreased ACC reward outcome responses to the blinded administration of glucose in participants who had recovered from bulimia. By contrast, Uher and colleagues [325] reported increased activation of the ACC, orbitofrontal cortex, occipital cortex, and cerebellum in response to food rewards in patients with bulimia; however, they did find hypoactivity in the lateral prefrontal cortex in patients with BN when compared with controls.

Several studies have included different patient groups relevant to eating disorders, allowing for identification of brain imaging patterns specific to different types of eating disorders. Schienle and colleagues [326] examined reward outcome by presenting food images to overweight and normal-weight controls, overweight individuals with binge-eating disorder, and normal-weight individuals with BN. These authors reported increased medial orbital frontal cortex activation in the binge-eating disordered group, and greater cingulate cortex and insula activation in the bulimic group, relative to all other groups. Brooks *et al*. [327] compared neural responses to food-reward outcomes in individuals with BN and with anorexia nervosa (AN), and found that individuals with BN had relatively greater activation in the dorsolateral prefrontal cortex, the insular cortex, and the pre-central gyrus. These studies compliment candidate genetic behavior investigations in BN that have reported altered allelic frequencies for the DAT gene [328] and DA receptor genes [329,330] in individuals with bulimia.

Molecular-imaging data addressing striatal DA function in BN are lacking. In the only preliminary study to be published, Tauscher and colleagues [331] reported a 15% reduction in striatal DAT availability in BN, although the study included only sub-threshold cases and an unmatched control group.

#### Dopaminergic treatments

Only fluoxetine, which primarily affects serotonin, is approved by the US FDA for the treatment for BN [332]. Of the numerous trials of the effects of psychopharmacologic agents for the treatment of BN, none has been primarily a DA agent [333].

##### Anorexia nervosa

AN is characterized by extremely low body weight, distorted body image, and fear of gaining weight, with an estimated prevalence of 0.7% [1,316]. Watson and colleagues [334] outlined a framework delineating linkages between AN and reward-processing deficits. Their model stressed the highly social nature of eating, the overlapping reward circuitry of gustatory and social stimuli [335,336], and the tendency of individuals with AN to deprive themselves of pleasure. Additionally, Zucker and colleagues [337] described commonalities between AN and ASD in social and interpersonal impairments, suggesting that impaired social function and social motivation may be a novel framework to conceptualize core deficits of AN.

#### Clinical studies

Individuals with AN report a heightened response to both punishment and reward outcome, even in the absence of clinically significant symptoms of anxiety or depression [338]. Fladung and colleagues [339] assessed responses to images depicting a female body with underweight, normal-weight, and overweight canonical whole-body features. They reported higher ventral striatal activation during processing of underweight images compared with normal-weight images in women with acute AN, but the reverse pattern in the control group. Joos and colleagues [340] also reported hyper-reactive reward-outcome responses in anorexia during the processing of food-reward images.

A small handful of studies have directly compared reward responses in AN and bulimia. Wagner and colleagues [341] reported increased CN activation to monetary-reward outcomes in women recovered from anorexia, and relatively equivalent CN responses to monetary gains and losses (a strongly similar pattern of results to that found by Wagner *et al*. in bulimia in [323]), suggesting possible similarities in reward-circuitry response in AN and bulimia. Uher and colleagues [325] also found similar brain-activation patterns in individuals with AN and bulimia, with both groups showing hyperactivation relative to controls in areas relevant to reward processing, including the ACC and the orbitofrontal cortex.

However, other studies have emphasized brain-activation differences during reward outcome between anorexia and bulimia. Brooks and colleagues [327] found that in response to food-reward outcomes, individuals with anorexia had greater activation of the dorsolateral prefrontal cortex, the cerebellum, and the right pre-cuneus relative to controls. They also had greater activation of the caudate, superior temporal gyrus, right insula, and supplementary motor area, and greater deactivation in the parietal lobe and dorsal posterior cingulate cortex relative to those with bulimia. It should be noted that this study did not include a non-food-reward condition, a design feature that would be necessary to assess the functional integrity of brain-reward systems to different classes of rewards.

Interestingly, individuals at risk for an eating disorder (that is, those with higher dietary restraint) have enhanced anticipatory responses to food rewards in the orbitofrontal cortex and the dorsolateral prefrontal cortex [342], suggesting that hyperactive functioning of anticipatory reward processing may be a risk factor for eating disorders. Complimenting these functional magnetic resonance imaging (fMRI) studies is a report of higher ^11^ C-raclopride binding potential in the ventral striatum in women who were recovered from AN, suggesting that DA activity is enhanced in this population [343], and significant relations between multiple DRD_2_ polymorphisms and AN [344].

##### Dopaminergic treatments

Psychopharmacologic treatments for AN have yielded only moderate success, and the majority of treatments are antidepressants that act primarily on non-DA systems [333]. A small number of double-blind trials have evaluated the effects of antipsychotics, with essentially non-significant effects [345-347].

### Neurodevelopmental disorders

#### Schizophrenia

Schizophrenia is a complex and debilitating disorder that typically emerges in late adolescence and early adulthood, and is characterized by hallucinations and delusions (positive symptoms), social withdrawal, alogia, and flat affect (negative symptoms), and cognitive disabilities [1], and has an estimated lifetime prevalence of 1% [348]. Anhedonia has been hypothesized to be a core feature of schizophrenia [349-351], and it has been suggested that individuals with high levels of social anhedonia are more likely to develop schizophrenia-spectrum disorders [352], although the link between anhedonia and the so-called schizophrenia prodrome has not been firmly established [353]. The centrality of incentive motivation deficits to schizophrenia is suggested by the long-standing hypotheses regarding the role of DA disturbances in the pathophysiology of the disorder [354-356].

##### Preclinical models

The DA hypothesis of schizophrenia suggests that excess DA transmission may be pro-psychotic, and originally gained support from pharmacological evidence that drugs that decrease DA activity (for example,, the phenothiazine neuroleptics) are antipsychotic, whereas drugs that promote DA activity (for example,, amphetamines) are psychotomimetic [76,357]. Indeed, the medications that have proven successful for treating schizophrenia/psychosis are drugs that primarily antagonize D_2_ receptors [76,358]; however, most clinically effective antipsychotics also exhibit a myriad of other actions that contribute to both therapeutic and side-effect profiles [359,360].

Current models of schizophrenia suggest that the disorder is due to both common and rare gene mutations, copy-number variations, and possibly epigenetic factors [361], all of which can affect multiple brain neurotransmitter systems and multiple risk genes [362-364]. Using pharmacological and genetic approaches, animal models have been developed for schizophrenia, which manipulate or alter mesolimbic DA transmission as a means to understand the disease and/or test therapeutic strategies.

In rodent models, hyperlocomotive behaviors and disruptions in the pre-pulse inhibition (PPI) response (a measure of sensorimotor gating) are generally viewed as being psychotomimetic, as both hyperlocomotion and disrupted PPI can be normalized and attenuated by antipsychotic medications [365]. However, no current behavioral paradigms truly capture the positive symptoms of schizophrenia (such as hallucinations and delusions). PPI is a cross-species measure that refers to the ability of a non-startling ‘pre-stimulus’ to inhibit the response to a startling stimulus [366]. There have been numerous reports of PPI deficits in patients with schizophrenia [367,368]; however, exactly which endophenotype in schizophrenia is manifested as disrupted PPI remains debated [365]. Swerdlow and colleagues [368] persuasively suggested that PPI deficits are a useful psychophysiological outcome for basic studies in humans and animals to probe neural circuitry and as a pharmacological screen. Indeed, PPI testing is commonly used in screening for potential antipsychotic drugs that act via antagonism of mesolimbic DA transmission. Studies in mice have indicated that administration of direct-acting DA agonists (such as apomorphine) and indirect DA agonists (such as cocaine) to mice disrupt PPI primarily via D_1_ receptors [369], whereas D_2_ receptors seem to modulate amphetamine-induced PPI deficits [370]. By contrast, both apomorphine-induced and amphetamine-induced PPI disruptions in rats are blocked by DA D_2_ antagonists [366]. In addition, normalizing PPI deficits in rodent models has enabled drug discovery for potential antipsychotic medications [371], some of which have proven successful in treating schizophrenia [368,372].

Mice lacking the DAT gene display markedly increased levels of DA in the mesolimbic system and striatum [373], that results in hyperlocomoter behaviors [373,374] and also deficits in PPI [375,376]. The DAT knockout mice phenotypes resemble amphetamine-like effects, and both hyperlocomotion and PPI deficits can be reversed with either D_1_ or D_2_ receptor antagonists [376], the atypical antipsychotics clozapine and quetiapine [377], various antidepressant drugs, and monoamine transporter inhibitors [378]. Thus, the DAT knockout mouse may be a useful animal model for predicting the efficacy of novel drugs for disorders such as schizophrenia that are characterized by a dysregulated limbic DA system.

In alignment with the DA hypothesis of schizophrenia, an increased level of striatal D_2_ receptors has been seen in patients with schizophrenia who are not on medication [78], which may result in D_2_ receptor supersensitivity in the ventral striatum contributing to psychosis [79]. Kellendonk *et al*. [80] attempted to model this D_2_ receptor elevation in genetically engineered mice, in which they transiently and selectively overexpressed D_2_ receptors in the striatum including in the caudate putamen, the NAc, and olfactory tubercle. It was found that 30% of striatal MSNs overexpressed these engineered receptors, thereby elevating the D_2_ receptor level to about 15% higher than that of normal mice. To study the behavioral consequences of D_2_ receptor upregulation in the striatum, the mice were analyzed using a battery of behavioral tasks, and were shown to have several abnormal cognitive phenotypes, including working-memory deficits, reversal-learning impairment and decreased social interactions. In a follow-up study, Li and colleagues [379] reported that this D_2_ receptor overexpression in the striatum causes an increase in the firing activity of layer V cortical pyramidal neurons, and also a decrease in both the frequency and amplitude of spontaneous inhibitory post-synaptic currents, indicating reduced inhibitory transmission in the prefrontal cortex. Taken together, the mouse model suggests that overexpression of D_2_ receptors (similar to that seen in some individuals with schizophrenia) will alter striatal MSN activity, resulting in dysregulated GABA transmission and inhibitory activity in the cortex [380]. Because a core symptom of schizophrenia is cognitive impairment (for example, deficits in working memory, attention, executive function), this mouse model may provide a link explaining how altered mesostriatal and mesolimbic DA receptors and DA transmission can alter cognitive processes in the frontal cortex, possibly by dysregulating circuit pathways that link connectivity between the striatum and pre-frontal cortex [381]. The reader is referred to other seminal reviews of schizophrenia animal models that highlight altered DA and reward-pathway transmission [382-385].

##### Clinical studies

Patterns of responses to rewards by patients with schizophrenia are complex. Patients report normal intrapsychic emotional experience, but communicate symptoms of anhedonia during structured interview [386]. Individuals with schizophrenia show diminished positive and negative emotions in response to emotional movie clips [387], food [388], and social exchange [389-391], even when taking medication [388]. However, individuals with schizophrenia also report similar or heightened subjective emotional experience [392], including in response to movie clips [393], pictures [394], food [395], and even odors [396].

In contrast to the mixed self-report and interview profiles of hedonic capacity in schizophrenia, psychophysiological studies of patients with schizophrenia indicate comparable or more exaggerated facial responsivity to positive and negative stimuli, assessed via facial electromyography [397,398], skin conductance [399,400], and affective modulation of the startle eyeblink response [401,402]. These lines of evidence suggest that schizophrenia is characterized by deficits in the expression of pleasant emotions but not in the experiential or physiological components of emotions [390].

Studies that have differentiated between reward motivation and reward outcome in schizophrenia have found mixed results. Although some studies have found that individuals with schizophrenia are impaired during reward motivation and outcome [403-407], others have not found a selective impairment in reward motivation [386,408]. This discrepancy may be attributable to different levels of symptom severity in the patients sampled, as there is some evidence that the severity of clinical symptoms is correlated with reward motivation and outcome processing in schizophrenia [386,403].

Behavioral studies of reward learning have reported that sensitivity to reward is intact in schizophrenia, but deficits are evident in rapid reward learning on the basis of trial-to-trial feedback, such as reversal learning, and in reward-related decision-making [406,409-413]. However, reward learning may be typical in schizophrenia over longer learning trials [410], and in individuals with less severe symptoms [414]. Overall, however, studies of reward learning in individuals with schizophrenia are consistent with the framework that patients with schizophrenia have intact hedonic responses but impaired motivation and reward representation, leading to a failure to motivate their behavior for rewards [415].

Neuroimaging studies of responses to rewards in schizophrenia generally suggest decreased NAc activation during monetary-reward anticipation (but see [416]for an exception) in both patients taking medication and patients not taking medication [417-420]. However, there is also evidence that these effects may be mediated by the predictability or certainty of rewards, as individuals with schizophrenia have reduced activation of the ventral striatum to unexpected reward outcomes, but have enhanced responses to expected rewards [421]. There is also evidence of inverse relations between negative symptoms and NAc activation during reward anticipation [416,420,422] and between lateral PFC activation during reward outcomes [420]. Waltz *et al*. [406] used computer simulations to show that the reward-processing deficits in schizophrenia are consistent with impaired functioning of DA. A handful of studies also suggest that striatal responses during monetary anticipation in schizophrenia are partially normalized by the antipsychotic, olanzapine [279,417,423] but not by other antipsychotics [418], suggesting that this neural signature may be a state, rather than trait, marker of schizophrenia. Finally, Grimm and colleagues [424] reported reduced striatal activation in schizophrenia to food cues when medication dose and weight were used as covariates, highlighting a possible mechanism underlying weight gain in schizophrenia.

Molecular-imaging evidence indicates dysregulated striatal DA function in schizophrenia [77]. A meta-analysis of 17 studies found significant elevation of striatal D_2_ receptors in patients with schizophrenia who were not being treated with medication, although no consistent clinical correlates of this pattern were evident [78]. Studies have also suggested an increased affinity of D_2_ receptors for DA in schizophrenia, that may produce a D_2_ receptor supersensitivity in the NAc contributing to psychosis [79]. Additionally, a PET study found higher synaptic DA concentrations in the ventral striatum in schizophrenia [425].

##### Dopaminergic treatments

First-line treatments for schizophrenia include DA D_2_ receptor antagonist agents that primarily treat so-called positive symptoms. First-generation compounds, such as chlorpromazine and haloperidol, work primarily as D_2_ receptor antagonists [358]. Second-generation, or ‘atypical,’ antipsychotics, such as clozapine (which has affinity for D_2_ and D_4_ receptors [426]), risperidone, olanzapine, and quetiapine, primarily affect DA and 5-HT systems, but with markedly reduced extrapyramidal side effects [427]. Finally, third-generation antipsychotics, such as aripiprizole, are partial D_2_ receptor agonists with high affinity and low intrinsic activity, and these drugs may act as ‘DA stabilizers’ because of their ability to stabilize, rather than simply upregulate or downregulate functional output of DA systems [428].

#### Attention-deficit hyperactivity disorder

ADHD is characterized by symptoms of inattention, hyperactivity, or impulsivity that produce impairment in cognitive, behavioral, and interpersonal domains [1] Although for many years ADHD was believed to be a disorder of childhood and adolescence, it is now recognized to occur also in adulthood [120].ADHD affects approximately 8 to 9% of school-aged children and 4 to 5% of adults [429-431]. ADHD is characterized by symptoms of age-inappropriate inattention, impulsiveness, and hyperactivity [1]. It disrupts academic and social development, and is associated with considerable psychiatric comorbidity [432], including impaired academic, occupational, and social functioning, increased rates of substance abuse and traffic accidents, and persistent neuropsychological impairments [433-436].

Dysregulated reward processing has been proposed as a central mechanism in prevailing theoretical models of ADHD [437,438]. The ‘DA transfer deficit’ theory of ADHD highlights altered phasic DA responses to cues that predict rewards, resulting in decreased conditioning to reward cues, blunted reward anticipation, weaker influence of rewards on behavior, and ultimately poorer behavioral control [438,439]. This model explains not only empirical brain-imaging data of reward processing in ADHD (reviewed below) but also the consequences of these processes on motivated behaviors.

Clinical genetics studies have indicated that multiple genes are important in the development of ADHD. Recent meta-analyses of candidate gene association studies have found consistent evidence of significant associations between ADHD and polymorphisms in several candidate genes that are almost exclusively involved in the regulation of dopaminergic and serotonergic transmission (including the dopamine transporter (DAT1) gene, the dopamine D_4_ receptor (DRD4) gene, the dopamine D_5_ receptor (DRD5) gene, the serotonin transporter (5-HTT) gene, the 5-hydroxytryptamine receptor 1B (HTR_1B_) gene, and synaptosomal-associated protein 25 (SNAP25) [440,441]. Among these, perhaps the most commonly replicated risk gene associations were reported for DAT1 and DRD4; however, even for these genes, substantial population heterogeneity is seen in ADHD.

##### Preclinical models

Animal models of ADHD are expected to show phenomenological similarities to the clinical condition and mimic aspects of the three core symptoms of the disorder; that is, hyperactivity, impulsivity, and impaired sustained attention [442]. In addition, proof of predictive validity of ADHD animal models often includes evidence of improved behavioral outcome after treatment with effective ADHD therapeutics, including stimulants such as methylphenidate and amphetamine (reviewed below) which increase DA transmission and levels by reuptake inhibition of monoamine transporters.

Two commonly used ADHD rodent models are the DAT transgenic knockout mouse model and the spontaneous hypertensive rat (SHR) model, both of which exhibit altered mesostriatal DA transmission and model some aspects of ADHD behavior. Mice lacking DAT have increased dopaminergic tone and represent a genetic animal model in which certain endophenotypes of ADHD can be recapitulated [443]. In DAT knockout mice, DA is cleared very slowly from the synaptic cleft, causing a five fold elevation of extracellular DA in the striatum (that is, a hyperdopaminergic state). DAT knockout mice have been suggested to model ADHD because they are hyperactive ([443,444]), have reduced extinction of responses in food reinforcement operant tasks [445], and also have impaired learning and memory [444,446]. However, DAT knockout mice provide an extreme model because only a mild reduction in midbrain DAT binding has been seen in human adolescents with ADHD [447], and the model also does not agree with several studies have that found increased DAT in the striatum of children and adults in ADHD [448,449]. Nonetheless, the DAT knockout mouse provides very useful information concerning the neurobiological consequences of impaired DAT function which present as ADHD-like behaviors.

The most widely studied rodent model of ADHD is the inbred SHR [450]. The SHR is a convincing model to study because these rats have been shown to display many behavioral characteristics apparent in ADHD, including poor performance in sustained attention tasks, hyperactivity, impulsivity, sensitivity to delay, and increased variation in performance of operant tasks [451-453]. Impulsivity is seen in SHR as an inability to inhibit a response during the extinction phase of an operant task, and an inability to delay a response in order to obtain a larger reward [452,454]. It seems that the SHR exhibits these ADHD-like behaviors due to a genetic alteration in the DAT gene. The SHR possesses a 160-bp insertion in the noncoding region upstream of exon 3 of the DAT gene [455], which is of significance because a variable number of tandem repeats in the 3′-untranslated region of the DAT gene has been associated with ADHD in several family studies [448,449,456]. DAT gene expression is transiently reduced in the SHR midbrain during the first month after birth, and increased in adult SHR compared with controls [457], which results in abnormal mesostriatal DA transmission in the rats during postnatal development, and possibly in adulthood [458]. In addition, several other animal models have informed ADHD research; many of these models have implicated mesolimbic DA transmission as a feature underlying ADHD-like behaviors. The reader is encouraged to see other comprehensive reviews [443,450].

##### Clinical studies

Etiological models addressing cognitive dysfunction in ADHD have focused on altered reward sensitivity [18,459,460], including diminished influence of reward on skills [460], now-versus-later decision-making [461,462], and altered sensitivities during reward learning [463]. Altered reward processes are mediated via alterations in DA and other catecholamine function in ADHD [438,464-467]. Individuals with ADHD display a range of reward deficits, including impaired behavioral modification to rewards [468]. A classic finding in childhood ADHD is hypersensitivity to reward delays (that is, "delay aversion" [462,469-474]), which is independent of inhibitory deficits [475] yet correlates with hyperactivity symptom severity [476]. The ‘dynamic developmental theory’ of ADHD put forth by Sagvolden and colleagues [453,477] hypothesizes that downregulated frontolimbic DA results in lower tonic DA, a steeper and shorter delay-of reward gradient, and ultimately increased impulsivity and slower extinction of impulsivity. Longer delays between a behavior and its consequence then result in relatively reduced effects of the consequence for exerting control over the behavior in ADHD [453,459].

Although individuals with ADHD may report enhanced reward outcome responsivity [478], behaviorally, individuals with ADHD display a range of motivational deficits, including impaired behavioral modification in response to rewards [468], enhanced motivation for larger but riskier rewards [479], and decreased motivation for social rewards [480].

Although the majority of fMRI studies in ADHD have focused on attentional processes, such as cognitive control and response inhibition [481-483], a smaller subset of studies have focused on reward processing. Such studies have direct conceptual linkages to the constructs of impulsivity and delay aversion that are core features of the disorder. These studies have shown decreased ventral striatum activation during monetary-reward anticipation [484-487], atypical orbitofrontal activation during monetary-reward outcome [487,488], and decreased DCN and amygdala activation during delayed reward outcome [485,489]. Children with ADHD have reduced NAc activity when anticipating monetary rewards [18,486,487], which is seen particularly in drug-naive children [490], in carriers of the DAT nine-repeat allele [491], and in response to seeking gains rather than avoiding losses [492]. This pattern is present during cues of both immediate and delayed rewards [485]. Plichta and colleagues [485] also found relations between striatal responsivity to immediate and delayed rewards and ADHD symptom severity. Furthermore, a negative correlation between NAc activation during reward motivation for a range of rewards (monetary, verbal feedback, and loss avoidance) and the number of reported ADHD symptoms was found in the general population [492]. Finally, a study by Wilbertz and colleagues [493] found decreased differentiation between high-incentive and low-incentive rewards in the medial orbitofrontal cortex and in physiological arousal in patients with ADHD that correlated with risky decision-making and delay-discounting.

Molecular-imaging studies in ADHD suggest that impaired frontostriatal activation to rewards in ADHD may be linked to altered DA transmission. Ernst and colleagues [494] found relatively higher right midbrain accumulation of ^18^ F-DOPA in children with ADHD, that was correlated with symptom severity, and a study of children with ADHD by Volkow *et al.*[120] found lower specific DA binding to DATs and to D_2_ and D_3_ receptors in the NAc, midbrain, and left caudate, with D_2_ and D_3_ receptor binding in the NAc, midbrain, caudate, and hypothalamus significantly related to inattention symptoms. A follow-up study showed that decreased DA binding to these receptors was also correlated with lower scores of self-reported motivation [495]. Levels of tonic and phasic DA have also been found to be lower in individuals with ADHD [453,496] whereas the density of DATs, which downregulate DA activity, is higher in ADHD [448,449].

#### Dopaminergic treatments

Methylphenidate is the most commonly prescribed medication for childhood ADHD, and has binding affinity for both the DA and norepinephrine transporters [497]. D-amphetamine is the major pharmacological ingredient in dextroamphetamine and lisdexamfetamine dimesylate, and both are believed to exert their therapeutic actions by enhancing the function of noradrenaline and DA [498]. Finally, bupropion has been used off-label for treating ADHD, yet it has been shown to have only very moderate efficacy for treating core ADHD symptoms [499,500].

#### Obsessive–compulsive disorder

OCD has an estimated prevalence of 1 to 3% [501], and is characterized by recurrent anxiety-provoking thoughts or impulses (obsessions), typically followed by repetitive ritualistic behaviors to relieve anxiety (compulsions) [502]. Although OCD is formally classified as an anxiety disorder, it has many phenotypic features resembling addictive behaviors, including tolerance and withdrawal-like behaviors, suggesting linkages between core symptoms and reward-circuitry processes. Indeed, it has been theorized that compulsive behaviors may persist at least in part due to the rewarding effects of anxiety-reduction that accompanies them [503], and that OCD should be labeled as a disorder of behavioral addiction rather than as an anxiety disorder in the DSM-V [504]. Although OCD is grouped here as a psychiatric disorder (rather than a neurodevelopmental disorder), it is important to note that it is commonly seen in both children and adults [505].

##### Preclinical models

Animal models of OCD have focused on studying obsessive-like behaviors related to grooming and repetitive movements. In mice, the neural substrate for the stereotyped grooming sequence (whisker grooming or coat grooming) lies in several brain regions including the brain stem and the striatum [506], where the striatum is thought to regulate the initiation and modulation of these grooming behaviors [507].

Profiles of DAT knockout mice indicate involvement of altered mesolimbic DA transmission in OCD. DAT knockout mice show an overall increased level of DA transmission, resulting in increased DA tone, hypermotoric activity, and overall increased movement [373]. The DAT knockout mice also have stronger and more rigid self-grooming patterns, with mutants displaying sequential super-stereotypy, evidenced by having more stereotyped and predictable self-grooming sequences [508]. Synapse-associated protein 90/PSD-95-associated protein (SAPAP)3 is a post-synaptic scaffolding protein at excitatory synapses, that is expressed at high levels in the striatum. An engineered genetic knockout of SAPAP3 in mice increases anxiety and compulsive grooming behaviors, leading to facial hair loss and skin lesions [509], providing a genetic animal model for OCD-like behaviors. SAPAP3-deficient mice have dramatically increased grooming bouts, and spend significantly more time self-grooming than their genetically normal littermates. Physiological studies indicate that the mutant mice have multiple deficits in the excitatory synapses of the striatal MSNs, including increased striatal excitatory and NMDA-dependent neurotransmission.

Also related to genetic mouse models of OCD are the Slitrk5 knockout mice. Genetic deletion of Slitrk5 in mice also results in excessive grooming associated with facial hair loss and skin lesions and with impaired corticostriatal neurotransmission [510]. Although evidence for a direct disruption in mesolimbic transmission in SAPAP3 and Slitrk5 knockout mice has not been reported, the altered excitatory transmission apparent in the striatum may also dysregulate the reward pathways.

##### Clinical studies

Behaviorally, patients with OCD show evidence of impaired reward learning [511] and impaired performance on gambling tasks that predict pharmacologic treatment response [512,513]. Despite the overlap of OCD and substance-use disorders in terms of phenotype, neurobiology, comorbidities, and neurochemistry [514], few empirical studies have directly assessed reward-system integrity in OCD. Using a monetary incentive-delay task, Jung *et al*. [515] found increased frontostriatal activation during monetary-reward outcome, and decreased lateral prefrontal and inferior parietal cortex activation during loss anticipation, but no group differences during monetary-reward anticipation. However, Figee and colleagues [516] did find relatively decreased NAc activity during reward anticipation in OCD, particularly in those patients with contamination fear. Finally, Pena-Garijo and colleagues [517] found that individuals with OCD had reduced activity in the ACC and the CN during a reward-learning task. Clearly, more research is needed in this area, in particular studies of mesolimbic responses to disease-relevant stimuli and studies of the relationships between brain function in response to reward stimuli and treatment outcomes.

A review of PET studies by Whiteside *et al*. [518] indicated differences in radiotracer uptake in the orbital gyrus and the head of the CN in patients with OCD, and a quantitative, voxel-level meta-analysis of functional MRI findings by Menzies and colleagues [519] reported abnormalities in the orbitofronto-striatal regions in OCD. A recent review and meta-analysis of *in vivo* imaging studies assessing striatal DA systems in OCD found evidence of reduced D_2_ receptor binding in the neostriatum and ventral striatum, and reduced D_1_ striatal receptor binding [520].

##### Dopaminergic treatments

Although the most widely prescribed agents to treat OCD are tricyclic antidepressants and selective serotonin reuptake inhibitors, these agents show a 40 to 60% inadequate response rate [521]. Recently, second-generation antipsychotic agents have shown benefit, either alone or as adjunctive therapy. Specifically, high-dose olanzapine [522], quetiapine [523], and risperidone [524] have all shown at least minimal clinical benefit relative to placebo treatment [525]. Finally, reports that the ventral striatum is an effective target for deep brain-stimulation treatment in OCD, particularly in patients identified as otherwise treatment-resistant, further implicates the mesolimbic DA system in OCD [526-528].

#### Autism spectrum disorders

ASDs affect up to 1% of the general population [529], and are characterized by a triad of symptoms that includes impaired communication, social impairments, and restricted and repetitive behaviors and interests [1].

##### Preclinical models

Studies aimed at modeling, in animals, the core phenotypes associated with ASDs have focused on studying social and repetitive behaviors in mice [530-532] and pair-bonding behaviors in the prairie vole (*Microtus ochrogaster*) [533,534]. Although there is no clinical evidence supporting disordered attachment profiles in autism, these rodent models may provide a bridge to define and translate the neurobiology of mammalian social behavior into a better understanding of ASD [535]. As discussed in greater detail below, there is compelling evidence that the mesolimbic DA pathway is altered in a mouse model of FXS, and that DA signaling in the NAc is important for social pair bonding in the prairie vole, suggesting that altered reward processing can influence social behaviors.

The study of social bonding in the prarie vole is one animal model that has informed preclinical studies relevant to ASDs. Prairie voles display characteristics associated with a monogamous lifestyle, including a lack of sexual dimorphism, biparental care of offspring, and the formation of pair bonds between males and females [536]. In the laboratory, male and female prairie voles show a robust preference to pair-bond and associate with a familiar partner, and the neurobiology and behavior of this social-bonding attachment has been studied extensively [534]. Vole pair bonds can be assessed by testing for partner preference, a choice test in which pair-bonded voles regularly prefer their partner to a conspecific stranger. Several studies have indicated that mesolimbic DA pathways regulate vole pair-bonding and social behaviors [537]. After extended cohabitation with a female, male voles show behaviors indicative of pair-bond maintenance, including selective aggression towards unfamiliar females. These voles also show a significant upregulation in NAc D_1_-like receptors, and blockade of these receptors abolishes the selective aggression of the males toward unfamiliar females [538]. Mating between voles can facilitate partner preference formation, and is associated with increased extracellular DA in the NAc. This partner preference can be blocked by microinjection of the D_2_ antagonist eticlopride into the NAc (but not the prelimbic cortex), whereas the D_2_ agonist quinpirole can facilitate formation of vole partner preferences [539]; decreasing NAc cAMP signaling probably underlies these effects of D_2_ receptors [540]. Interestingly, in a comparative study of monogamous versus promiscuous voles, the monogamous voles exhibited increased mesolimbic DA release into the NAc in response to amphetamine, suggesting increased DA release or clearance in the monogamous species [541]. However, when amphetamine or a selective D_1_ receptor agonist was administered systemically to male voles, this interfered with mating-induced pair bonding, and this disruption seems dependent on D_1_ receptor activation [542-544]. Taken together, these prairie-vole social-bonding studies indicate that the mesolimbic DA is essential for the social pair-bond with D_2_-like receptors in the NAc.

Other neurotransmitter systems seem to converge within the NAc to modulate and control vole social-bonding behavior. For example, activation of cortisol-releasing factor (CRF) receptors by microinjections of CRF directly into the NAc accelerates partner preference formation in male prairie voles [545]. Oxytocin transmission and oxytocin receptors in the NAc seem to facilitate maternal behavior in female voles [546]. Furthermore, prairie voles have higher densities of NAc oxytocin receptors than is found in nonmonogamous vole species, and blocking NAc oxytocin receptors prevents partner-preference formation [547]. These studies suggest that oxytocin facilitates affiliation and social attachment [548].

Although the challenge remains to translate these observations and mechanisms from rodents to human autism studies [549], these animal model findings have had a significant influence on our understanding of mammalian social behaviors, and have generated testable hypotheses about the reward system and underlying molecular neurobiology. The reward-system involvement in social engagement and social-bond formation may also have implications for understanding the core social deficits characterizing ASDs [535].

##### Clinical studies

A number of theorists have suggested that the social-communication deficits that characterize ASDs reflect decreased motivation to engage in reciprocal social behaviors in infancy and early childhood, which may ultimately result in fewer experiences with social sources of information [550-552]. Because children with ASD may lack the motivation to participate in activities in which social skills are typically forged, the resulting relatively impoverished social environment may further compound the social impairment caused by low social motivation, and further negatively influence the development of social cognition and language skills [553,554]. Consistent with this model, very young children with ASD display decreased orienting to social stimuli [550,555], and atypical social orienting has been shown to predict decreased social competence in adolescents and young adults with ASDs [556]. There is also evidence that social motivation remains impaired in individuals with ASD despite growth in other areas of cognitive development. For instance, older children with ASDs report experiencing less pleasure from social rewards [557], and social stimuli are relatively less salient for individuals with ASD [558-560]. More generally, individuals with ASD have been found to report lower levels of reward responsivity [561], and behavioral studies have also found evidence for impaired reward learning in individuals with ASD [562].

However, despite the accumulating evidence for reward-processing deficits in ASD, relatively few published studies have assessed the neural bases of reward processing in this population, and results of these studies are decidedly mixed. Schmitz and colleagues [563] investigated the neural substrates of reward learning in the context of a sustained attention task with monetary rewards, and reported decreased activation in the left anterior cingulate gyrus and left midfrontal gyrus on rewarded trials in patients with ASD. They also found that activity in the anterior cingulate gyrus during this task was negatively correlated with social ability, supporting the hypothesized link between that reward-processing dysfunction and the core social impairments in ASD. Scott-Van Zeeland and colleagues [564] investigated the neural correlates of implicit reward learning in children with ASDs using both social and monetary rewards. They found diminished ventral striatal response during social-reward outcomes and also, but to a lesser extent, with monetary-reward outcomes. Activity within the ventral striatum was found to predict social reciprocity within the control group but not the ASD group. This finding is consistent with previous research examining the effect of reward type on task performance, which indicates that children with ASD may be less motivated by social than non-social rewards [480,565-568]. Dichter and colleagues [569] recently reported results of an fMRI study of reward anticipation and outcome using monetary and social (faces) rewards within the context of an incentive-delay task. The ASD group displayed bilateral amygdala hyperactivation during face-reward anticipation and bilateral insular cortex hyperactivation during face-reward outcomes. Further, activation in the left and right amygdala during face anticipation predicted the severity of social impairments in the ASD sample.

EEG and event-related potential (ERP) studies have largely supported these fMRI findings. Kohls and colleagues [570] examined responses in children with ASDs during a rewarded go/no-go paradigm involving social (smiling faces) and monetary rewards, using an ERP marker of reward-system activity. In the ASD group, they found unimpaired behavioral task performance but a decreased response to reward conditions that required an active response for both social and monetary rewards A recent EEG study found evidence of relatively decreased left-sided frontal EEG activity in response to faces, a pattern suggestive of decreased motivational approach [571,572]) By contrast,, Larson and colleagues [573] reported that an ERP marker of reward processing was unimpaired in ASD, highlighting the need for future research to examine differential brain activation to reward gains and losses in ASD.

Restricted and repetitive behaviors and interests are also a core symptom of ASDs, and a number of etiologic models of repetitive behaviors highlight that reward-processing deficits may bias attention and exploration towards non-social aspects of the environment [552,559]. This general behavioral tendency may ultimately lead to the development of stereotyped movements and circumscribed interests that characterize ASDs [574]. To investigate the hypothesis that reward-processing dysfunction in ASD may contribute to the development of circumscribed interests, Dichter and colleagues [575] conducted a fMRI study in which stimuli reflecting circumscribed interests were presented to individuals with ASDs within the context of an incentive-delay task. The ASD group showed decreased NAc activation during monetary-reward anticipation, but ventromedial prefrontal cortex hyperactivation when viewing the circumscribed interest reward outcome, suggesting that ASDs are characterized by reward-circuitry hypoactivation in response to monetary incentives but by hyperactivation during circumscribed interest reward outcomes.

Manipulating the consistency, immediacy, or saliency of rewards has been a central feature of long-standing effective behavioral interventions for ASD [576-578]. Such programs are designed to scaffold reward understanding for children with ASD to ultimately alter behavior and enhance learning. Even with this scaffolding, however, reward-based interventions are not successful for all children with ASD [579-582], and there is some evidence that individual differences in reward motivation may predict differences in response to treatment [580]. Such variability suggests an urgent need to identify neurobiological markers to aid in the prediction of responses to reward-based behavioral interventions in ASD, and to understand how these markers may be functionally related to behaviors relevant to treatment success.

Aberrant serotonin function has consistently been linked to genetics, neuropharmacology, and brain metabolism of individuals with ASD [583]. There are few studies of striatal DA binding in ASD, and to date there has been no consistent evidence of striatal DAT-binding differences in ASD [584,585], although a recent study with a relatively large sample found evidence of higher DAT binding in the orbitofrontal cortex in ASD [586]. Ernst and colleagues [587] found reduced ventromedial prefrontal cortex DA metabolism in children with ASDs, whereas Nieminen-von Wendt and colleagues [588] found no such evidence. A small pilot study of 13 children with ASDs who received a 6-month course of fluoxetine treatment showed that good clinical responders had a significant decrease in striatal DAT binding [589], suggesting that studies of modulation of striatal DAT binding may be relevant to understand potential mechanisms of action of treatments for ASD, even when such treatments do not primarily affect DA systems.

##### Dopaminergic treatments

Although SSRIs have been a promising class of agents to target repetitive behaviors in ASD [590-594] (but inefficacy has also been reported [595]), the only two drugs currently approved by the US FDA for the treatment of ASDs are the second-generation antipsychotic risperidone (a DA antagonist) and the third-generation antipsychotic aripiprazole (a D_2_ partial agonist). Although both are approved for the treatment of irritability, an associated ASD symptom, both have shown efficacy in reducing core symptoms as well. Specifically, randomized controlled trials of risperidone in individuals with ASD found significant reductions in challenging behaviors, such as irritability and hyperactivity [596-600], and significant improvement in core autism symptoms [601-604]. Randomized controlled trials of aripiprazole have also found a decrease in irritability and hyperactivity and decreased instances of repetitive behaviors in children with ASD over the course of treatment [604,605].

Other commonly used treatments for ASD include other antipsychotic agents [606-608], psychostimulants (for example, methylphenidate), which generally upregulate norepinephrine and/or DA function and reduce hyperactivity but have a relatively poor side-effect profile [609,610], and naltrexone, a DA modulator [607,611].

#### Tourette’s syndrome

TS affects 0.3 to 0.8% of the population [612], and is characterized by motor and vocal tics (rapid, recurrent, stereotyped motor movements or vocalizations) performed in response to somatosensory or environmental cues [1]. This defining feature of TS suggests involvement of nigrostriatal DA motor control systems, and thus it is not unexpected that striatal systems linked to reward processing have been implicated in the disorder, through not using tasks assessing response to rewards.

##### Preclinical models

Animal models relevant to TS have focused on rodent genetic models and behavioral phenotypes such as stereotypy. The DAT knockout mouse is one model involving hyperdopaminergia that results in increased levels of DA in striatal brain regions, which may model some of the motor abnormalities apparent in TS. Excessive sequential stereotypy of behavioral patterns (sequential super-stereotypy) in TS is thought to involve dysfunction in the nigrostriatal DA systems, and DAT knockout mice exhibit complex restricted patterns of stereotyped movements similar to the sequential super-stereotypy seen in TA [508]. The genetic factors underlying TS are largely unknown; however, a rare mutation in the gene *SLITRK1* is associated with human TS [613,614]. The SLITRK1 protein is a single-pass transmembrane protein that displays similarities to the SLIT family of secreted ligands, which have roles in axonal repulsion and dendritic patterning in neurons, but its function and developmental expression remain largely unknown. A SLITRK1 knockout mouse model of TS has recently been developed. SLITRK1 knockout mice exhibit increased anxiety-like behavior in the elevated plus-maze test and neurochemical analyses identified increased levels of norepinephrine and its metabolite 3-methoxy-4-hydroxyphenylglycol in the prefrontal cortex and NAc, but DA levels were not altered [615]. Administration of clonidine, an α2-adrenergic receptor agonist often used to treat patients with TS, attenuated the anxiety-like behavior of SLITRK 1-deficient mice, providing predictive validity in this TS mouse model. Interestingly, SLITRK 1 expression in mouse, monkey, and human brain is developmentally regulated in the neuroanatomical circuits most commonly implicated in TS [616]. In the striatum, SLITRK 1 expression is high in striosomes/patches during early brain development but significantly diminishes later, suggesting a possible role in establishing corticostriatal circuitry. In addition, SLITRK 1 expression is also restricted to striatal projection neurons of the direct pathway where it could influence striatal circuitry; however, to date, direct evidence of SLITRK 1 knockout affecting either mesolimbic or nigrostriatal DA pathways has not been reported.

##### Clinical studies

The DA hypothesis of TS was originally proposed nearly 30 years ago [617], and has been corroborated by post-mortem data [618]; however the identification of an underlying DA deficit leading to dysfunction in TS has proven to be elusive [619]. Available evidence suggests that a phasic DA imbalance, similar to that seen in schizophrenia, may help to explain the pathophysiology of TS [619,620]. Supporting this framework, reward learning is enhanced in people with TS who are not on medication, and impaired in people with TS taking DA receptor antagonists [621].

Although there are no published functional neuroimaging studies of response to rewards in TS, a recent fMRI study of tic inhibition implicated the striatum and associated dorsal frontal regions during tic suppression [622], corroborating other evidence that the magnitude of basal ganglia and thalamus activation during voluntary tic suppression correlated inversely with the severity of tic symptoms [623]. Thus, a model has been proposed in which frequent prefrontal activation during tic suppression may produce compensatory prefrontal cortex hypertrophy that aids in tic suppression [624,625], although it is not presently clear how such basal ganglia and prefrontal characteristics effects reward processing in TS. Variants in the DA receptor gene *DRD*_*2*_ have also been associated with genetic risk for TS [626-628], but this finding is not consistent [629-633].

An early study of monozygotic twins discordant for TS severity found evidence in affected twins of increased D_2_ receptor binding in the head of the CN, but not putamen, which predicted disease severity [634]. Single-photon emission computed tomography investigations in TS have found higher DAT binding in the right caudate [635], the striatum [636-639], the putamen after amphetamine challenge [640], and the basal ganglia [641]. However, a handful of studies have found no differences in striatal DAT binding in TS [639,642-644].

##### Dopaminergic treatments

Tetrabenazine is commonly used to treat hyperkinetic movement disorders, including TS [645]. Its mechanism of action is believed to involve the early metabolic degradation of monoamines, in particular DA [646]. The classes of medications with the most proven efficacy in treating tics are typical and atypical antipsychotics (DA receptor antagonists) including risperidone, ziprasidone, haloperidol, pimozide, and fluphenazine [647].

#### Conduct disorder/oppositional defiant disorder

CD is defined by a behavioral pattern involving the violation of others’ rights and of societal rules along with antisocial behaviors before the age of 18 years, and ODD is characterized by recurrent patterns of defiant behaviors toward authority figures during childhood [1]. These externalizing disorders, known collectively as disruptive behavior disorders, are often comorbid, and there is debate over whether they represent differences in severity of symptom expression or two distinct conditions [648]. Prevalence estimates for both conditions are just over 3% [649]. Although the preponderance of functional brain-imaging studies in these conditions has focused on cognitive switching and sustained attention [650], a significant subset of studies has focused on reward processing.

##### Clinical studies

Results of functional brain-imaging studies of response to rewards in CD/ODD are not wholly consistent [17]. Rubia and colleagues [651] reported reduced orbitofrontal activation during a rewarded continuous performance task in adolescents with CD, whereas Bjork *et al*. [652] reported increased subgenual cortex activation in adolescents with externalizing disorders during a monetary incentive-delay task. Finally, Crowley and colleagues [653] found that adolescents with CD and comorbid substance-use disorder displayed relative hypoactivation in the striatum and ACC during risky decision-making for rewards.

Despite evidence that the dopaminergic system plays a key role in aggression [654,655], only a small handful of molecular genetic studies implicate DA candidate genes, including DAT1, DRD_2_, and DRD4, in the development of conduct problems [656-659], and no molecular-imaging study to date has assessed striatal DA signaling in samples with CD/ODD who are not comorbid for other conditions.

##### Dopaminergic treatments

Atypical antipsychotics, psychostimulants, mood stabilizers, and α2 agonist agents are commonly used to treat CD/ODD [660]) Divalproex was found it to be superior to placebo in treating explosive temper, mood lability [661], and CD [662] in adolescents. There is initial evidence in the form of open-label or retrospective chart review studies, of the efficacy of olanzapine, quetiapine, and aripiprazole in treating aggressive behavior [663]. Risperidone was found to be well-tolerated and superior to placebo in reducing aggressive behaviors in children with CD [664-666]. Two large controlled trials found risperidone to be superior to placebo in ameliorating hostile and aggressive behavior in lower-functioning children with disruptive behavioral disorders [667,668].

### Genetic syndromes

#### Prader-Willi syndrome

PWS is characterized by infantile hypotonia, mental retardation, short stature, hypogonadism, hyperphagia and early-onset morbid obesity [669]. It has an estimated prevalence of 1 in 10,000 to 1 in 30,000 births [670]. Approximately 70% of cases are due to a genetic deletion on chromosome 15 (15q11–13), 25% of cases are due to a maternal uniparental disomy of chromosome 15, and the remaining cases result from imprinting defects [671,672]. Although to date there are no preclinical models of PWS that clearly implicate the mesolimbic reward system, a linkage between PWS and reward-processing deficits is suggested by hyperphagia (abnormally increased appetite for and consumption of food) and the high incidence of obesity in affected individuals. PWS is the most commonly recognized genetic cause of childhood obesity, and obesity is the primary basis of morbidity and mortality for individuals with the syndrome. If given access, individuals with PWS will consume three to six times as much food as individuals without the syndrome, and show delayed meal termination, and earlier return of hunger after a previous meal [673,674]. Children with PWS show enhanced behavioral responses to food cues, which do not diminish after receiving a favorite food [675], suggesting that the incentive salience of food is heightened in this population, and that this heightened motivation is not diminished with satiation [676]. Appetite disturbance in PWS has been attributed to the hypothalamic dysfunction that characterizes the disorder, which also causes growth-hormone deficiency, hypogonadism, and temperature dysregulation [677].

The enhanced response to food that characterized PWS suggests that the brain reward-circuitry response to food may be hyperactive. Indeed, several research groups have found greater activation in the ventromedial prefrontal cortex, amygdala, and orbitofrontal cortex during reward anticipation for food cues in PWS. Miller and colleagues [678] presented images of food, animals, and tools, and found that participants with PWS had relatively greater ventromedial prefrontal cortex activation to food compared with controls. Holsen and colleagues [679] scanned participants with PWS while they viewed images of food and animals, both before and after eating a standard meal. They found a group × time interaction, reflecting the increased activation in orbitofrontal cortex, medial prefrontal cortex, and insula at post-meal relative to pre-meal in the PWS groups that was not evident in the control group. Dimitropoulos and Schultz [680] reported increased activation in the hypothalamus and orbitofrontal cortex in PWS during high-calorie versus low-calorie food-reward outcomes.

These initial studies suggest that the hyperphagia in PWS may indeed be mediated by hyperactivation in brain-reward networks to food-related stimuli (but Hinton *et al*. [681] failed to replicate this finding). In support of these findings, Shapira and colleagues [682] showed that in patients with PWS that there was a temporal delay in response to glucose ingestion in the resting-state activity of a distributed network implicated in the regulation of hunger and satiation, namely the hypothalamus, insular cortex, ventral basal ganglia, and ventromedial prefrontal cortex.

To date there are no published molecular-imaging studies addressing striatal DA binding in PWS, and the first-line pharmacologic treatment for PWS is growth-hormone therapy, which does not regulate DA function.

#### Williams syndrome

WS (also known as Williams–Beuren syndrome), with a prevalence of about 1 in 7,500 [683], is a neurodevelopmental condition caused by a hemizygous microdeletion on chromosome 7q11.23, and is characterized by hypersociality and being overly empathic [684,685]. Although to date there are no preclinical models of WS that clearly implicate the mesolimbic reward system, the highly social phenotype of WS suggests a poorly modulated reward-system response to social cues.

There are currently three models of social function in WS: 1) heightened drive towards non-specific social interaction (for example, social interactions with strangers) [686,687], 2) heightened emotional responsiveness [685], and 3) social fearlessness [688]. Only two functional brain-imaging studies to date to have provided insight into reward processing in WS by assessing responses to happy and fearful faces. One such study found relations between amygdala responses to fearful faces specifically, and symptoms of social approach of strangers [689], supportive of the social fearlessness model. Another study found that individuals with WS show reduced amygdala activity in response to sad faces and comparable activity in the orbitofrontal cortex for happy and sad faces, whereas TS individuals show a heightened orbitofrontal cortex response to sad faces [690]. However, both of these studies did not report responses to the happy-face conditions. In this regard, it is currently unknown if the phenotype of hypersociality in WS reflects a hyperactive neurobiological response to social rewards specifically, or if such behavior is but one exemplar of indiscriminant heightened approach behaviors to a wider range of social-emotional stimuli.

There are no published studies of striatal DAT function in WS. WS is typically treated by anxiolytic and antipsychotic agents, but to date there are no systematic data on efficacy of DA agents to treat WS [691].

##### Angelman syndrome

AS is characterized by intellectual disability, epilepsy, impaired coordination, and absence of speech [692]. However, individuals with AS also commonly exhibit a characteristic happy demeanor with prominent smiling, non-specific laughing, a general exuberance [693], and an attraction to water and certain types of paper and plastics [694], suggesting that the reward-system function may be a candidate system for study in this syndrome [695]. The syndrome is caused by mutations or deletions of the maternal copy of the gene *Ube3a*, an E3 ubquitin ligase enzyme that is involved in targeting proteins for degradation in cells. Although empirical evidence for altered reward-system function in individuals with AS is lacking, animal models suggest there may be some involvement. In elegant genetic studies using the fruit-fly *Drosophila*, overexpression or genetic knockout of the *Drosophila* homolog of *Ube3a* respectively increased or decreased DA levels, potentially due to changes in the expression of tetrahydrobiopterin, the rate-limiting cofactor in monoamine synthesis in flies [696]. In addition, in a mouse model of AS, genetic loss of the *Ube3a* gene resulted in a loss of DA neurons in the substantia nigra [697], which may contribute to mechanisms that cause ataxia and motor deficits apparent in the mouse model of the disease. The severe developmental delay that characterizes the syndrome renders functional neuroimaging research challenging, and to date there are no functional brain-imaging data on responses to rewards or molecular brain-imaging studies in AS.

##### Rett syndrome

RS predominantly affects females and is caused by mutations in the gene encoding the methyl-CpG binding protein (MeCP)2, a transcriptional repressor involved in DNA remodeling and regulation of gene expression. In RS. both loss of function and gain in MeCP2 gene dosage lead to similar neurological phenotypes [698]. MeCP2 mutations result in a number of pathologies including microencephaly, general growth retardation, motor clumsiness, ataxia, and autistic features, including social withdrawal, loss of language, and stereotypy [699].

##### Preclinical models

Although a connection between dysregulated mesolimbic DA reward systems and RS is not currently clear, several studies indicate altered DA levels and changes in the closely related nigrostriatal DA pathways in mouse models. It has been suggested that MeCP2 protein normally functions in the NAc to limit the rewarding properties of psychostimulants, and that psychostimulant or DA receptor-induced phosphorylation of MeCP2 may be involved in the rewarding properties of drugs of abuse such as amphetamines [700]. In an engineered MeCP2-deficient mouse model, a postnatal reduction of DA and its metabolite homovanillic acid was seen in the caudate putamen [701,702], suggesting that MAO and/or COMT levels might be impaired. Loss of MeCP2 also compromises the nigrostriatal DA pathway in mice, where the number of DA-synthesizing neurons is significantly decreased in the substantia nigra of RS model mice [701]. In addition, the DA neurons in the substantia nigra of MECP2 mutant mice have a decreased capacitance, total dendritic length, and resting membrane conductance as early as 4 weeks after birth, well before overt neurodevelopmental symptoms are seen in the mouse model [703]. These studies suggest that nigrostriatal DA deficits may underlie the origin of motor dysfunctions in RS. Although further studies are required, there may also be similar deficits in the closely associated mesolimbic pathway in RS model mice.

##### Clinical studies

Because of the profound cognitive impairment associated with RS [699], there are no functional brain-imaging studies of individuals with this condition because of the cognitive demands of the functional brain-imaging environment. Molecular-imaging studies have indicated increased D_2_ receptor binding in the caudate and putamen [704], and in the striatum as a whole [705]. No DA agents are first-line treatments for RS.

#### Fragile X syndrome

FXS is the most common inherited cause of intellectual disability, occurring in 1 in 4,000 males and 1 in 8,000 females [706], and is caused by a mutation of the *FMR1* gene on the long arm of the X chromosome(locus Xq27.3; [707]). The *FMR1* full mutation affects cognition, adaptive behavior, social abilities, and motor skills [708]. Specific areas of cognitive weaknesses include communication, mathematics, visual-spatial processing, executive function, and memory [709-711].

##### Preclinical models

Fragile X model mice have been developed that encode an engineered mutation in the *FMR1* gene [712], recapitulating the human mutation, resulting in an absence of FMR1 expression. To identify potentially related neurochemical mechanisms affected by this mutation, Fulks and colleagues [713] used fast-scanning cyclic voltammetry to measure electrically evoked DA release in striatal brain slices. In adult mice, a decrease in stimulated extracellular DA release and reuptake was seen in FMR1 mutant mice, which was also associated with decreased repetitive movements/stereotypy. FMR1 has been suggested as important for DA signaling in both the prefrontal cortex and striatum, where it may interact with G protein–coupled receptor kinase 2, which regulates DA receptor signaling cascades [714]. In studies of FMR1 mutant mice, Zhuo and colleagues reported that cortical and striatal neurons from the mutant mice exhibit abnormal D_1_ receptor signaling and disrupted synaptic plasticity in response to D_1_ receptor activation. Remarkably, these neuronal deficits could be rescued by restoring the *FMR1* gene to the mutant neurons [715]. FMR1 is also suggested to be important for D_1_ receptor-mediated synthesis of SAPAP3 in prefrontal cortex neurons; SAPAP3 is a post-synaptic neuronal scaffolding protein that regulates glutamate receptor trafficking and function [716]. Together, these studies indicate that FMR1 mutant mice have dysregulated striatal DA transmission and pre-frontal DA receptor function, and that these changes may contribute to the mechanisms underlying FXS.

##### Clinical studies

No functional brain-imaging studies to date have assessed responses to rewarding stimuli in FXS. Rather, given that individuals with FXS display social impairments that may be similar to those seen in ASD [717,718], it is not surprising that functional brain-imaging studies of FXS have focused almost exclusively on responses to social stimuli. To the extent that, in neurotypical development, social stimuli such as faces are rewarding [719], these studies may indirectly address the integrity of reward-circuitry function in FXS, although future studies designed to investigate striatal response to other rewards in FXS are needed.

Garret and colleagues [720] reported relatively decreased activation of the fusiform gyrus and superior temporal sulcus and increased right insula activation to images of faces in FXS. The authors suggested that these results may reflect anxiety provoked by the face stimuli. Watson, Hoeft, Garrett *et al*. [721] showed this same stimulus set to boys with FXS, and replicated the finding of greater insula activation (although on the left side) to direct gaze. Holsen *et al*. [722] reported decreased cingulate and left insula activation in individuals with FXS in response to images of familiar fearful faces and inverse relations between social anxiety and activation in a number of regions, including a cluster in the left inferior frontal gyrus near the insula. These authors speculated that social anxiety in FXS has a cascading effect on multiple aspects of cognition. Finally, Dalton *et al*. [723] compared responses in groups with FXS and with autism during the processing of emotional faces, and found that in response to faces, the FXS group had higher activation in the right insula (among other regions) compared with both the autism and control groups, a finding that the authors suggested may be linked to social anxiety.

The only published molecular-imaging study of striatal DAT binding is a small investigation of four patients with parkinsonism carrying the FXS permutation, which found initial evidence of decreased striatal binding [724]. However, there is evidence that DA functioning may be atypical in FXS, as shown by high rates of comorbidity with tremor disorders [725], higher blink rates [726], and emerging preclinical models [714].

##### Dopaminergic treatments

Children and adults with FXS are regularly prescribed stimulants, antidepressants, anticonvulsants, and antipsychotics [727-729]. Psychostimulants are the most often prescribed psychoactive medication to treat FXS [730], with initial randomized controlled trial data of response to methylphenidate and dextroamphetamine suggesting moderate response rates on attention and social skills [731]. Preliminary studies of aripiprazole in FXS have also found evidence for an improvement in clinical symptoms and irritability [732,733].

## Conclusions

The central tenet of this review is that multiple neurodevelopmental and psychiatric disorders and genetic syndromes share a common neurobiological characteristic, namely, altered functional output of striatal DA systems mediating the processing of rewards. This framework suggests the need for new methods of phenotypic assessment that cut across traditional symptom-based surveys developed to assess functioning based on traditional, category-based classification systems such as *DSM*[1] and the *International Classification of Diseases*[734]. Given that a significant portion of the disorders reviewed here respond favorably to treatment by psychopharmacologic agents that primarily affect DA systems, investigations of reward-circuitry functioning in psychopathology may have direct relevance not only for etiological models of disease mechanisms, but for the potential mechanisms of effective interventions and the development of treatment agents. Although treatment effects do not necessarily indicate pathophysiological etiologies, the efficacy of dopaminergic agents represents supportive preliminary evidence of a potential common etiology in a number of conditions.

However, we recognize that the one-to-one linking of mesolimbic DA function with reward response is clearly overly simplistic [45]. The mesolimbic DA system represents only one component of a very complex and integrated set of circuits, and although DA is clearly a crucial neurotransmitter in the reward-processing system, non-DA systems clearly also modulate reward responsibility [19,735]. Additionally, there are multiple brain regions not addressed in this review that contribute to reward processing, including the subthalamic nucleus and ventral pallidum, the subiculum, the lateral habenula, and the extended amygdala [736,737]. Additionally, multiple non-DA compounds have shown efficacy in improving reward responses, such as agents that affect glutamate circuits involved in regulating monoamine systems [19]. Consequently, the purpose of this review is to serve as a starting point for consideration of DA-mediated reward-system dysfunction as a potential common etiologic factor in a range of conditions. Future research aimed at understanding linkages between disease phenotype, reward function, and treatment response will clearly have to consider other interacting systems and neurotransmitters.

A host of unanswered questions remain about how ascending DA projections and their forebrain targets contribute to aspects of reward processing [738,739]. For example, even within the context of DA systems, it is not clear whether increased and decreased reward-oriented motivation is a result of decreased or increased sensitivities of DA and associated systems [735,740]. Moreover, the striatum and associated DA systems play a prominent role in processing aversive stimuli and processing rewards [741,742]. Hence, the present review is intended to highlight initial evidence of the relevance of DA reward to neurodevelopmental and psychiatric disorders, but clearly future studies are needed to address other brain circuits, neurotransmitters, and motivating stimuli.

Another limitation of this line of research is that the majority of clinical studies summarized in this review assessed responses to standardized rewards, such as money or standard picture sets. This approach relies on the implicit assumption that standardized stimulus sets are a reasonable proxy for individual-specific stimuli. In the realm of nonclinical cognitive neuroscience, this assumption seems to be valid [97]; however, the concordance of results using standardized versus individual-specific stimuli is largely unknown in clinical contexts. Additionally, there are a number of contexts in which reward-system dysfunction in a given disorder may be contingent on a particular class of stimuli (for example,, addiction cues in substance abuse [148], food images in eating disorders [326], and sad pictures in unipolar depression [232]). Although this issue complicates cross-disorder comparisons, this variability in response-eliciting stimuli leads to distinct phenotypic expressions in different disorders.

Future research is needed to delineate linkages between laboratory measures of reward processing and real-life experiences of incentive motivation, positive affect, reward-seeking, and risk-taking tendencies. The few studies that have evaluated potential relations between mesolimbic neural activity and subjectively experienced reward [220] or motivation to work for rewards [154] have yielded promising initial results suggesting the external validity of laboratory-based measures of reward processes, but research on the ecological validity of reward-processing endophenotypic measures is needed. Additionally, the development of measures sensitive to reward-system integrity and suitably sensitive to change for intervention studies are also needed. Although self-report [743-745] and behavioral [209,746] measures of reward capacity have been developed, their association to neurobiological function has proven to be limited. As suggested by Treadway and Zald [19], an implication of clinical neurobiological research into reward-system dysfunction may be the modification of psychiatric interviews to frame and code questions to tap hedonic capacity and motivation towards certain classes of stimuli.

Another area in need of greater research is reward-circuitry function in comorbid disorders. Given that the conditions reviewed here share mesolimbic dysfunction, it is perhaps not surprising that there are high rates of comorbidity between these disorders. For example, there are high rates of comorbidity between substance abuse and other Axis I conditions [747-749], schizophrenia and bipolar disorder [750], and ASD and mood and anxiety disorders [751]. A number of explanations for the high comorbidity rates have been suggested, including shared genetic etiology, self-medication of symptoms, and common socioenvironmental determinants [752,753], but only multi-group studies that directly compare cases with comorbid disorders will be able to distinguish the nature of reward-circuitry dysfunction in these contexts.

Finally, as previously noted, the vast majority of clinical research into reward-circuitry function is cross-sectional in nature, and has focused only on adults. Given the importance of brain development prior to adulthood, the study of reward-related processes during development will be crucial to disambiguate the proximal effects of altered reward-circuitry function from its more downstream effects on learning, motivation, and overall functioning [754-756]. There may be critical periods during early development when mesolimbic dysfunction creates a predisposition to any number of disorders, and understanding the factors that mediate these processes will be essential for treatment and the prevention of symptom onset.

## Competing interests

The authors declare that they have no competing interests.

## Authors’ contributions

All three authors were major contributors in writing this manuscript, and have read and approved the final manuscript.
